# Erythropoietin regulates developmental myelination in the brain stimulating postnatal oligodendrocyte maturation

**DOI:** 10.1038/s41598-023-46783-9

**Published:** 2023-11-09

**Authors:** Paola Muttathukunnel, Michael Wälti, Mostafa A. Aboouf, Christina Köster-Hegmann, Tatjana Haenggi, Max Gassmann, Patrizia Pannzanelli, Jean-Marc Fritschy, Edith M. Schneider Gasser

**Affiliations:** 1 Institute of Pharmacology and Toxicology, University of Zürich, 8057 Zürich, Switzerland; 2 Center for Neuroscience Zurich (ZNZ), Zurich, Switzerland; 3 Institute of Veterinary Physiology, Vetsuisse Faculty, University of Zürich, 8057 Zurich, Switzerland; 4 Zurich Center for Integrative Human Physiology (ZIHP), University of Zurich, 8057 Zurich, Switzerland; 5 Department of Biochemistry, Faculty of Pharmacy, Ain Shams University, Cairo, 11566 Egypt; 6 Rita Levi Montalcini Center for Brain Repair, University of Turin, 10126 Turin, Italy

**Keywords:** Developmental biology, Molecular biology, Neuroscience

## Abstract

Myelination is a process tightly regulated by a variety of neurotrophic factors. Here, we show—by analyzing two transgenic mouse lines, one overexpressing EPO selectively in the brain *Tg21*(*PDGFB-rhEPO*) and another with targeted removal of EPO receptors (EPORs) from oligodendrocyte progenitor cells (OPC)s (*Sox10-cre;EpoRfx/fx mice*)—a key function for EPO in regulating developmental brain myelination. Overexpression of EPO resulted in faster postnatal brain growth and myelination, an increased number of myelinating oligodendrocytes, faster axonal myelin ensheathment, and improved motor coordination. Conversely, targeted ablation of EPORs from OPCs reduced the number of mature oligodendrocytes and impaired motor coordination during the second postnatal week. Furthermore, we found that EPORs are transiently expressed in the subventricular zone (SVZ) during the second postnatal week and EPO increases the postnatal expression of essential oligodendrocyte pro-differentiation and pro-maturation (*Nkx6.2* and *Myrf*) transcripts, and the *Nfatc2/calcineurin* pathway. In contrast, ablation of EPORs from OPCs inactivated the Erk1/2 pathway and reduced the postnatal expression of the transcripts. Our results reveal developmental time windows in which EPO therapies could be highly effective for stimulating oligodendrocyte maturation and myelination.

## Introduction

CNS developmental myelination comprises a series of cellular processes including the proliferation of oligodendrocyte precursor cells (OPCs), their differentiation into mature myelinating oligodendrocytes, and neuronal axon myelin ensheathment^1^. The system is mainly controlled by multiple transcription factors^2^; molecular determinants including growth factors, hormones, cytokines, surface receptors, and secreted ligands^3^; and axonal activity^4^. Hypoxia (low oxygen supply) and/or ischemia (low blood supply) cause severe disturbance of myelination in preterm infants^5^ and animal models of hypoxic brain injury^6^, mainly due to alterations in OPC maturation. Several studies have identified the hormone erythropoietin (EPO) in neuroprotection after perinatal brain injury^7–10^. In fact, EPO activates anti-inflammatory, anti-apoptotic, and proliferating pathways, and promotes neuronal and oligodendrocyte development following hypoxic-ischemic CNS injuries^7,8,10–16^. EPO stimulates oligodendroglia differentiation and myelination^16–19^ by driving myelin gene transcription such as myelin oligodendrocyte glycoprotein (MOG), myelin basic protein (MBP)^17^, and inducing genes involved in lipidic transport and metabolism^20^, making EPO a potential pharmacological agent that counteracts demyelinating diseases or perinatal white matter injury^21–23^. EPO administration in preterm infants still presents inconsistencies with regard to neurodevelopmental outcomes^24^. Therefore, identification of the temporal expression of the EPO receptors (EPORs) in specific cell types during development and after hypoxia–ischemia is essential to optimize the therapeutic schemes.

During early embryonic development, EPO and EPOR expression reaches blood-like levels in the neural tube^5^. EPO then markedly decreases to persist at low levels through adulthood^25^. Using a transgenic mutant mice overexpressing human EPO only in the brain under the platelet-derived growth factor B promoter (*Tg21, PDGFB-EPO*)^26,27^, we have previously shown that EPO accelerates the neuronal maturation of GABAergic and glutamatergic neurons in the hippocampus^28^. We also showed that EPO activates the extracellular signal-regulated kinase 1/2 (Erk1/2) and AKT pathways in neurons, stimulates brain mitochondrial function, increases mitochondrial mass and vesicle numbers in the synaptic terminals and enhances cognition^29^. EPO-mediated Erk1/2 activation, GABAergic signaling, and mitochondrial function allegedly influence the initiation of myelination^30–32^. However, it is still unknown whether, during postnatal brain development, EPO is also a signaling pathway for stimulating oligodendrocyte maturation and myelination. Here, we evaluated in mice, the postnatal expression of EPOR in the subventricular zone (SVZ) of the lateral ventricle, an area of oligodendrogenesis, and found a very high postnatal expression. Thus, we aimed to investigate the role of EPO in developmental myelination and its regulation in oligodendrocyte progenitor cell (OPC) proliferation and differentiation. We analyzed two transgenic mutant mice, the *Tg21,PDGFB-EPO* overexpressing EPO preferentially in neuronal cells without alterations in systemic EPO expression^26,27^, and one with targeted ablation of EPORs from OPCs (*Sox10-cre;EpoR*^*fx/fx*^)^33^.

## Results

### EPO overexpression in the brain promotes postnatal brain growth and developmental myelination

**Figure 1:**
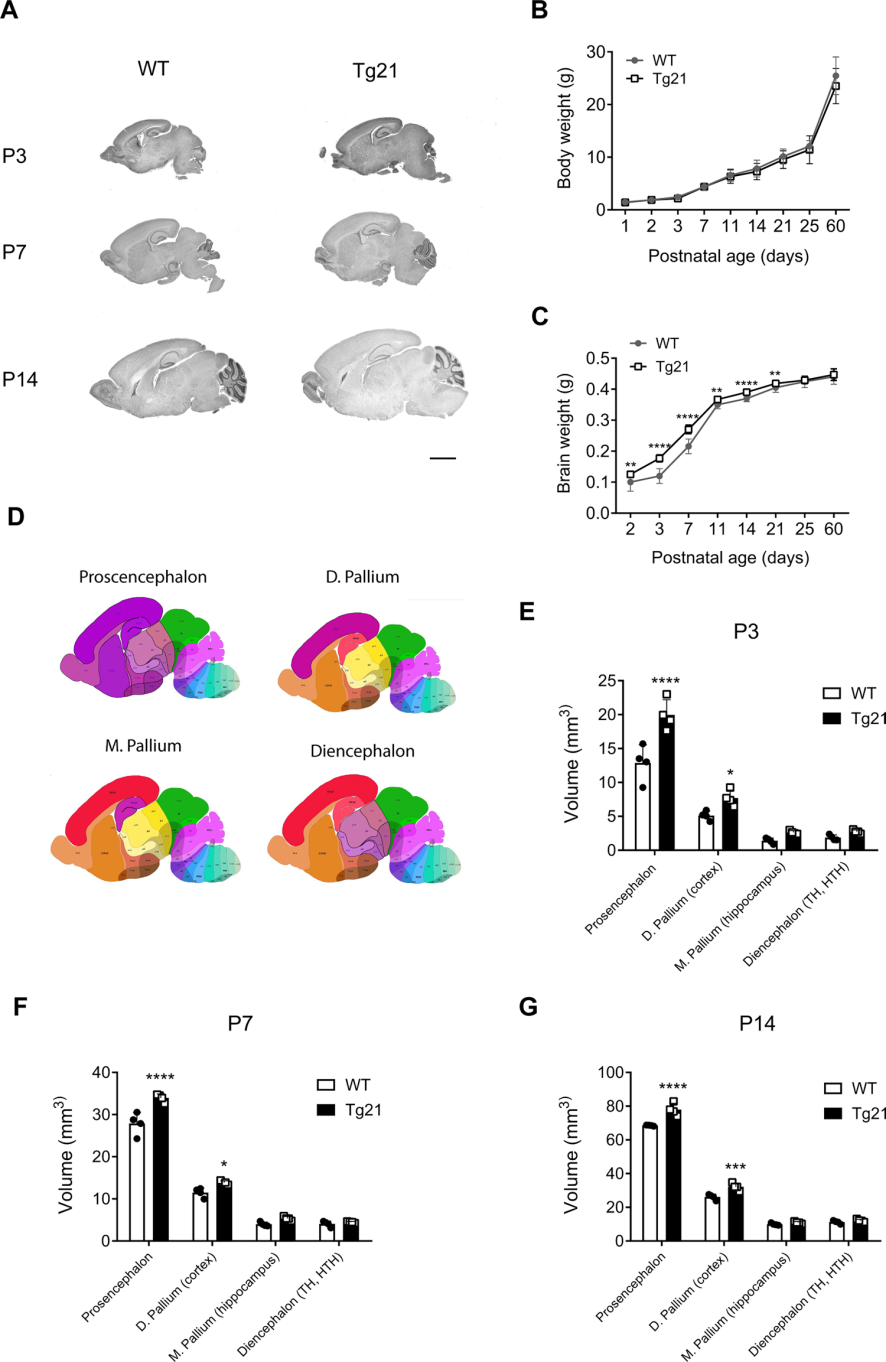
Larger postnatal brain volume in Tg21 mice. (**A**) Representative bright field sagittal brain images of hematoxylin and eosin staining (H&E) label in WT and Tg21 mice at postnatal ages (P): 3, 7, and 14. Scale bar: 1 mm. (**B**) Body weight across development in WT and Tg21 mice. Data are given as mean ± SD, WT (N_P1-2_ = 10, N_P3_ = 15, N_P7_ = 12, N_P11_ = 18, N_P14_ = 56, N_P21_ = 37, N_P25_ = 17 and N_P60_ = 15) and Tg21 (N_P1-2_ = 10, N_P3_ = 15, N_P7_ = 8, N_P11_ = 26, N_P14_ = 44, N_P21_ = 25, N_P25_ = 14 and N_P60_ = 27). Tg21 mice are slightly lighter after P21. 2-way ANOVA, F(1,333) = 5.6, P = 0.023. (**C**) Brain wet weight across development in WT and Tg21 mice. Data are given as mean ± SD, N equal as B. Greater brain weight is observed in Tg21 mice during postnatal development. 2-way ANOVA, F(1,333) = 178, P < 0.0001. Multiple comparisons: **P < 0.01, ****P < 0.0001. (**D**) Areas (labeled in Lila) in which the total volume analysis was made by stereology. (**E–G**) Volumes of brain areas across development in WT and Tg21 mice showing larger volumes in Tg21. N = 4 animals per age and genotype. (**E**) Brain volumes at P3. 2-way ANOVA, F(1,24) = 37.95, P < 0.001. Multiple comparisons: *P = 0.047, ****P < 0.0001. (**F**) Brain volumes at P7. 2-way ANOVA, F(1,24) = 41.01, P < 0.001. Multiple comparisons: *P = 0.027, ****P < 0.0001. **G**) Brain volumes at P14. 2-way ANOVA, F(1,24) = 50.8, P < 0.001. Multiple comparisons: ***P = 0.0002, ****P < 0.0001.

We first evaluated body and brain postnatal growth from all our WT (N = 180) and Tg21 (N = 169) experimental male animals (Fig. 1). We noticed no significant effect of the genotype on body weight gain across postnatal ages P1 to P14. However, starting at P21, Tg21 mice were slightly lighter (Fig. 1B, the 2-way ANOVA, F(1,333) = 5.196, *P* = 0.023). Nevertheless, brain weight was significantly larger in Tg21 mice across postnatal development and equal to controls at P25 and P60 (Fig. 1C, the 2-way ANOVA, F (1,333) = 178, *P* < 0.0001). The impact of EPO on postnatal brain weight was also reflected by a larger postnatal brain volume (Fig. 1A,E–G). Analysis of total volumes (V_tot_) of the *prosencephalon*, *d. pallium* (cortex), *m. pallium* (hippocampus) and *diencephalon* (TH and HTH) (areas represented in lilac Fig. 1D), revealed larger volumes in *prosencephalon*, *d. pallium* (cortex) in Tg21 mice at P3 (Fig. 1E, the 2-way ANOVA, F(1,24) = 37.95, *P* < 0.001); P7 (Fig. 1F, the 2-way ANOVA, F(1,24) = 41.01, *P* < 0.001); and P14 (Fig. 1G, the 2-way ANOVA, F(1,24) = 50.8, *P* < 0.001). No differences in brain volume were observed at P25 and P60.

**Figure 2:**
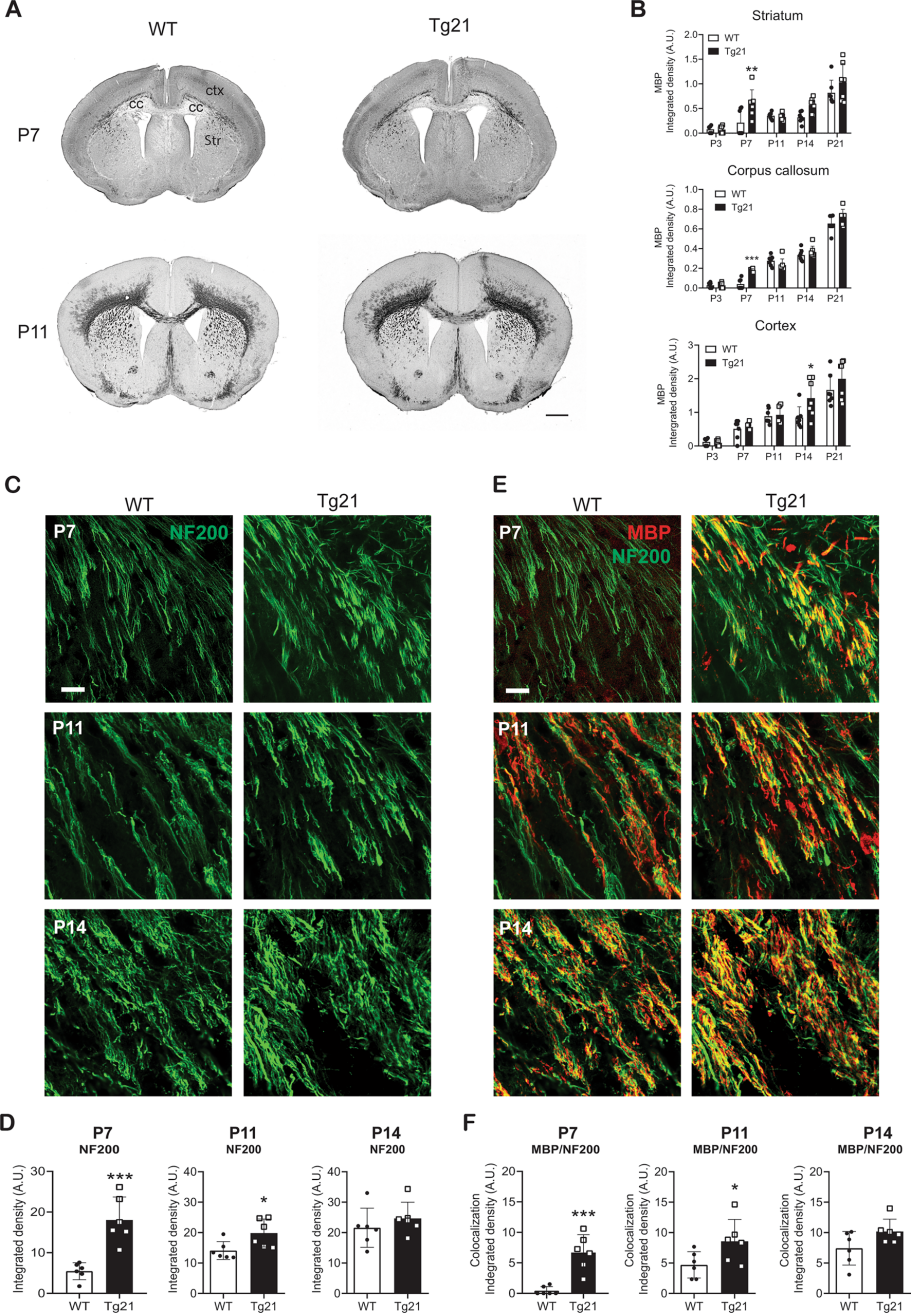
Earlier onset of myelination in Tg21 mice. (**A**) Representative coronal images of MBP staining at P7 and P11 show an increase in myelination with age and stronger staining in the Tg21 striatum and corpus callosum at P7. WT P7 image shows the areas of myelination analysis: ctx = cortex; cc = corpus callosum and Str = striatum. Scale bar: 1 mm. (**B**) Densitometric analysis of MBP expression in the striatum, corpus callosum, and cortex across postnatal ages P3, P7, P11, P14 and P21. Data are given as mean ± SD, N = 7 animals per age and genotype. Striatum: 2-way ANOVA, F(1,60) = 11.42, P = 0.0013. Multiple comparisons: **P = 0.0033. Corpus callosum: 2-way ANOVA, F(1,60) = 5.599, P = 0.0025. Multiple comparisons: ***P = 0.0004. Cortex: 2-way ANOVA, F(1,60) = 2.64, P = 0.0085. Multiple comparisons: *P = 0.013. (**C**) Representative fluorescent confocal images of the axonal marker heavy neurofilament (NF200, green) in the striatum of WT and Tg21 mice at postnatal ages P7, P11, and P14. Scale bar: 20 μm. (**D**) Integrated density of NF200 immunoreactivity in WT and Tg21 mice. P7, unpaired t-test, ***P = 0.0005. P11, unpaired t-test, *P = 0.027. P14, unpaired t-test, P = 0.39. N = 6 animals per age and genotype. (**E**) Representative fluorescent confocal images of myelin (MBP, red) and the axonal marker heavy neurofilament (NF200, green) in the striatum of WT and Tg21 mice at postnatal ages P7, P11, and P14. A higher myelination ratio observed by co-localization (yellow), occurs in the Tg21 striatum at P7 and P11. Scale bar: 20 μm. (**F**) Myelination axonal coverage (MBP/NF200) in WT and Tg21 mice. P7, unpaired t-test, ***P = 0.0005. P11, unpaired t-test, *P = 0.041. P14, unpaired t-test, *P* = 0.179, N = 6 animals per age and genotype.

Next, myelin basic protein (MBP) expression was assessed in coronal brain sections from WT and Tg21 mice at postnatal ages (P): 3, 7, 11, 14, and 21 (Fig. 2A,B). MBP immunoreactivity was analyzed in the striatum, corpus callosum, and cerebral cortex. At P3, MBP was undetectable in both genotypes. At P7, it became weakly discernable in WT mice, whereas it was visible in Tg21 mice, notably in the striatum (Fig. 2B, upper panel, 2-way ANOVA, F(1,60) = 11.42, *P* = 0.0013) and corpus callosum (Fig. 2B, middle panel, 2-way ANOVA, F(1,60) = 5.599, *P* = 0.0025), pointing towards earlier onset of myelination. In the cerebral cortex, a stronger MBP immunoreactivity was observed in Tg21 than in WT at P14 (Fig. 2B, lower panel, 2-way ANOVA, F(1,60) = 2.64, *P* = 0.0085).

During development, axons accumulate neurofilaments independently of myelin formation^34^. We investigated whether EPO influences the accumulation of neurofilament and/or axonal myelination by double immunostaining of heavy neurofilaments (NF200) and MBP in the striatum, an area where axons run in parallel and can be easily distinguished for analysis, at postnatal ages P7, P11 and P14 (Fig. 2C–F). Quantification of axonal density (represented by image fraction covered by immunoreactive NF200 axons) revealed a significant effect of both age (2-way ANOVA, F(2,30) = 17.13, *P* < 0.0001) and genotype (2-way ANOVA, F(1,30) = 20.21, *P* < 0.0001) (Fig. 2C,D). At P7, more NF200 immunoreactivity was seen in Tg21 mice (unpaired t-test, *P* = 0.0005, df = 10, Fig. 2D left graph). At P11, the genotype effect was less strong, but higher axonal density was still observed in Tg21 mice (unpaired t-test, df = 10, *P* = 0.027, Fig. 2D middle graph). No differences in axonal density were observed at P14 (unpaired t-test, df = 10, *P* = 0.39, Fig. 2D right graph). Next myelination axonal coverage (MBP/NF200) was evaluated (Fig. 2E,F). At P7, no myelinated axons were seen in WT mice, but in Tg21 mice, many axons were already myelinated (unpaired t-test, df = 10, *P* = 0.0005, Fig. 2F left graph). At P11, a smaller fraction of myelinated axons was seen in WT compared to Tg21 mice (unpaired t-test, df = 10, *P* = 0.041, Fig. 2F middle graph). No differences in axonal myelination were observed at P14 (unpaired t-test, df = 10, *P* = 0.179, Fig. 2F right graph). Increase in NF200 and MBP coverage in Tg21 at P7 and P11, suggests an effect of erythropoietin on accelerating developmental axonal density, and myelination.

**Figure 3:**
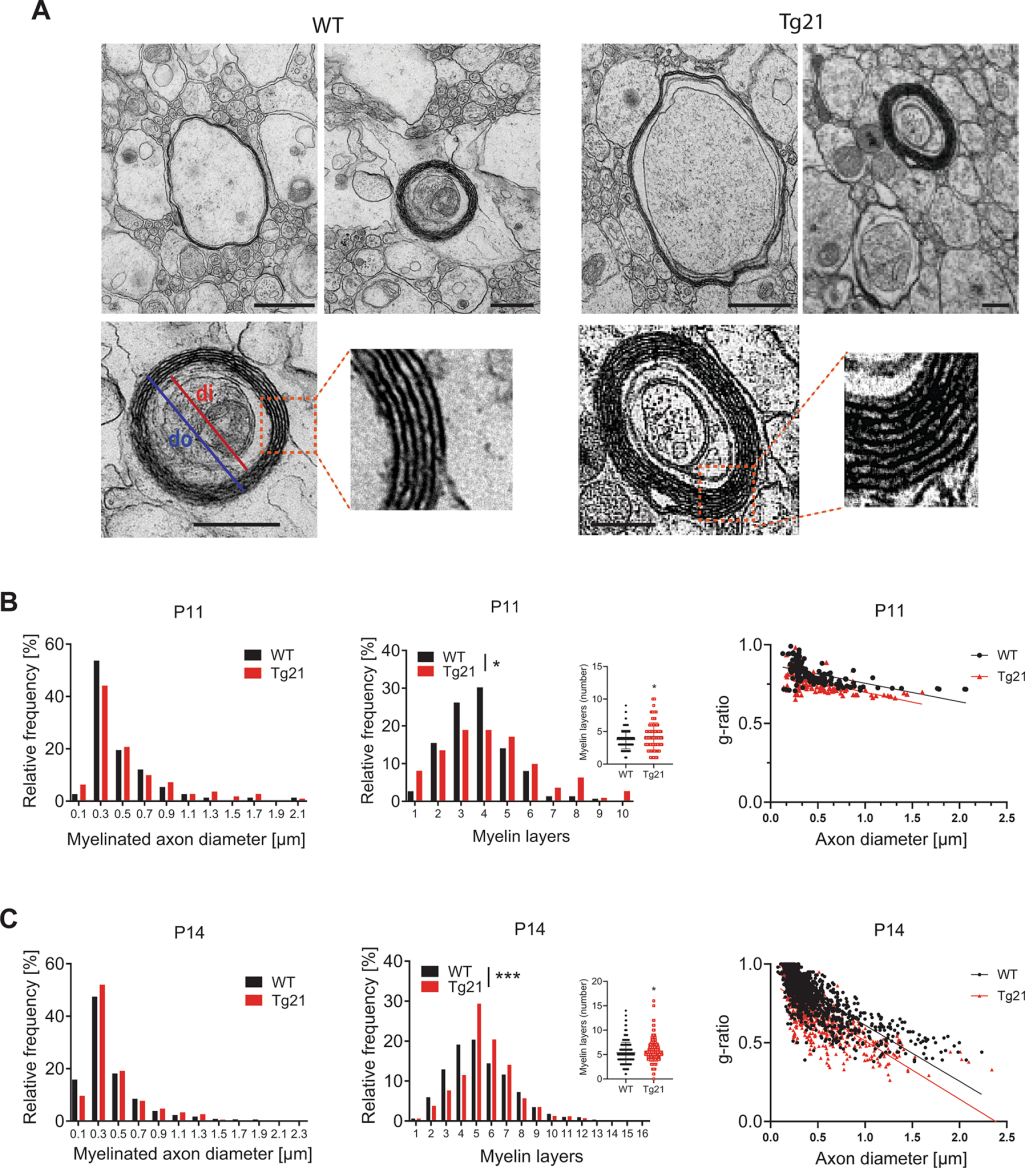
Ultrastructural analysis of developmental myelination showing increased axonal ensheathment in Tg21 mice. (**A**) Representative electron microscopy ultrastructural images from corpus callosum in WT and Tg21 mice at P11. The lower panels show enlargement of myelinated axons in which a major number of myelin layers was counted per genotype. Scale bar: 0.5 μm. (**B**) Relative frequency distribution graphs of myelinated axon diameters (left), myelin layers (middle), and myelin thickness (g-ratio) (right) in WT and Tg21 mice at P11. 2-sample KS test, *P = 0.04. Insets showing data points distribution. Unpaired t-test, *P < 0.05, N = 260. (**C**) Relative frequency distribution graphs of myelinated axon diameters, myelin layers, and myelin thickness (g-ratio) in CC from WT and Tg21 mice at P14. 2-sample KS test, ***P < 0.001. Insets showing data points distribution. Unpaired t-test, *P < 0.05, N = 2580.

To further investigate the impact of EPO on myelination, we performed an ultrastructural analysis in the white matter at P11 and P14 (n = 6 mice/group), focusing on the initial formation of the myelin sheath around callosal axons. At P11, 150 myelinated axons in WT and 111 in Tg21 mice were analyzed. At P14, 1464 myelinated axons in WT and 842 in Tg21 were analyzed. Although the total number of myelinated axons could not be determined for this area because numerous profiles could not be identified unambiguously, an almost ten-fold increase in the number of myelinated axons was quantified at P14 in a similar number of sections. The frequency distribution analysis of myelinated axonal diameters showed no correlation between axonal diameter and myelin sheaths nor difference across genotypes at either age (Fig. 3B,C, left panels, 2-sample KS: 0.1998 and KS: 0.218). A genotype difference was seen in the number of myelin layers at P11 and P14, with a larger fraction of axons having more myelin layers in the Tg21 mice (Fig. 3B,C; middle panels, KS-Test, *P* = 0.04, and *P* < 0.001), suggesting that the formation of the myelin sheaths occurs faster in Tg21 mice than in WT mice. The enhanced number of layers in Tg21 is also reflected by thicker myelin layers, as shown by the reduced g-ratio (Fig. 3B,C, right panels). These results corroborate that EPO overexpression influences the myelin formation rate, accelerating ensheathment around the axons.

### EPO stimulates early postnatal oligodendrocyte precursor cells maturation and activates the Erk1/2 pathway

**Figure 4:**
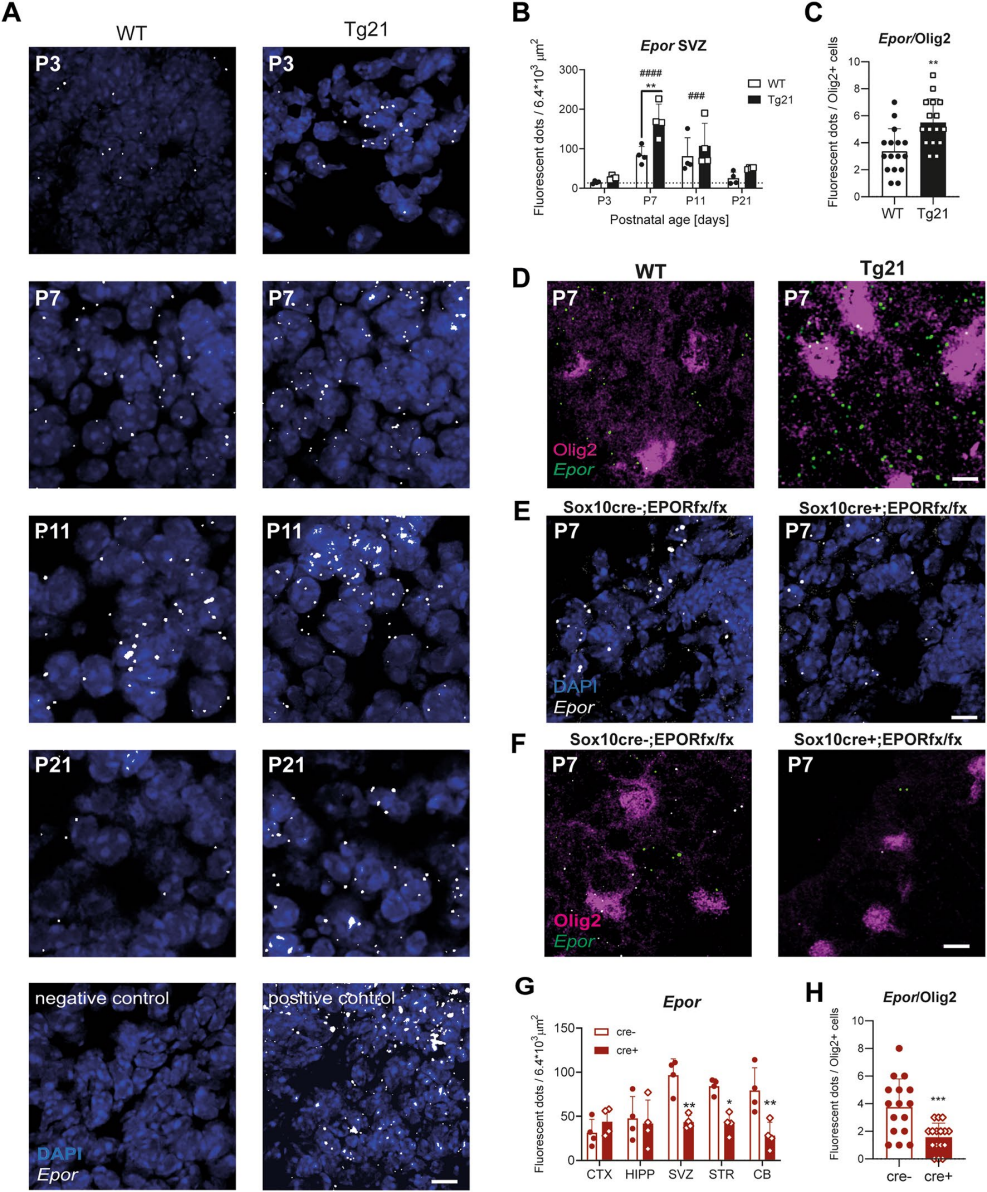
High *Epor* expression in the lateral ventricles' subventricular zone (SVZ) during postnatal development. (**A**) Representative confocal images of FISH for mRNA *Epors* co-labeled with DAPI in WT and Tg21 mice at P3, P7, P11, and P21 in the SVZ. Negative control: fluorophore; positive control: housekeeping gene. Scale bar: 10 μm. (**B**) Quantification of *Epor* mRNA fluorescent dots in SVZ at different postnatal ages, showing its high expression at postnatal day 7 (P7) and 11 (P11) in both genotypes. More *Epor* mRNA dots are quantified at P7 in Tg21 mice. Two-way ANOVA ages, F(3,24) = 19.7, ****P < 0.0001. Multiple comparison: P7 vs P3 and vs P21, ^####^*P* < 0.0001; P3 vs P11, ^###^*P* = 0.0006. Two-way ANOVA WT vs Tg21, F(1,24) = 11.2, **P = 0.0027. Multiple comparisons, **P = 0.0027. N = 4 animals per genotype and age. (**C**) Quantification of *Epor* mRNA dots in the Olig2+ cells from the SVZ from WT and Tg21 mice at P7. Each data point represents the average number per cell for one analyzed field of view. Three analyzed images per animal. Unpaired t-test, **P = 0.0070, N = 4 animals per genotype, (**D**) Representative confocal images of FISH for mRNA *Epors* and Olig2 immunofluorescence staining in the SVZ at P7 in WT and Tg21 mice. Scale bar: 10 μm. (**E**) Representative confocal images of FISH for mRNA *Epor*s co-labeled with DAPI in the SVZ from *Sox10-cre*^+*/*+^*;EpoR*^*fx/fx*^ (cre-) and *Sox10-cre*^*Tg/*+^*;EpoR*^*fx/fx*^ (cre +) animals at P7. Scale bar: 10 μm. (**F**) Representative confocal images of FISH for mRNA *Epors* and Olig2 immunofluorescence staining in the SVZ from *Sox10-cre*^+*/*+^*;EpoR*^*fx/fx*^ (cre-) and *Sox10-cre*^*Tg/*+^*;EpoR*^*fx/fx*^ (cre +) animals at P7. Scale bar: 10 μm. (**G**) Quantification of *Epor*s mRNA dots at P7 in different brain areas from Cre− (control) and Cre + mice. 2-way ANOVA, F(1,30) = 23.64, ****P < 0.0001. Multiple comparisons: SVZ **P = 0.0014, STR *P = 0.0124, CB **P = 0.002. N = 4 animals per genotype. Abbreviations: CTX: cortex, HIPP: hippocampus, SVZ: subventricular zone, STR: striatum, CB: cerebellum. (**H**) Quantification of *Epor* dots in the Olig2+ cells from the SVZ in Cre− and Cre+ mice at P7. Each data point represents the average cluster number per cell for one analyzed field of view. Four analyzed images per animal. Unpaired t-test, **P = 0.0017. N = 4 animals per genotype.

**Figure 5:**
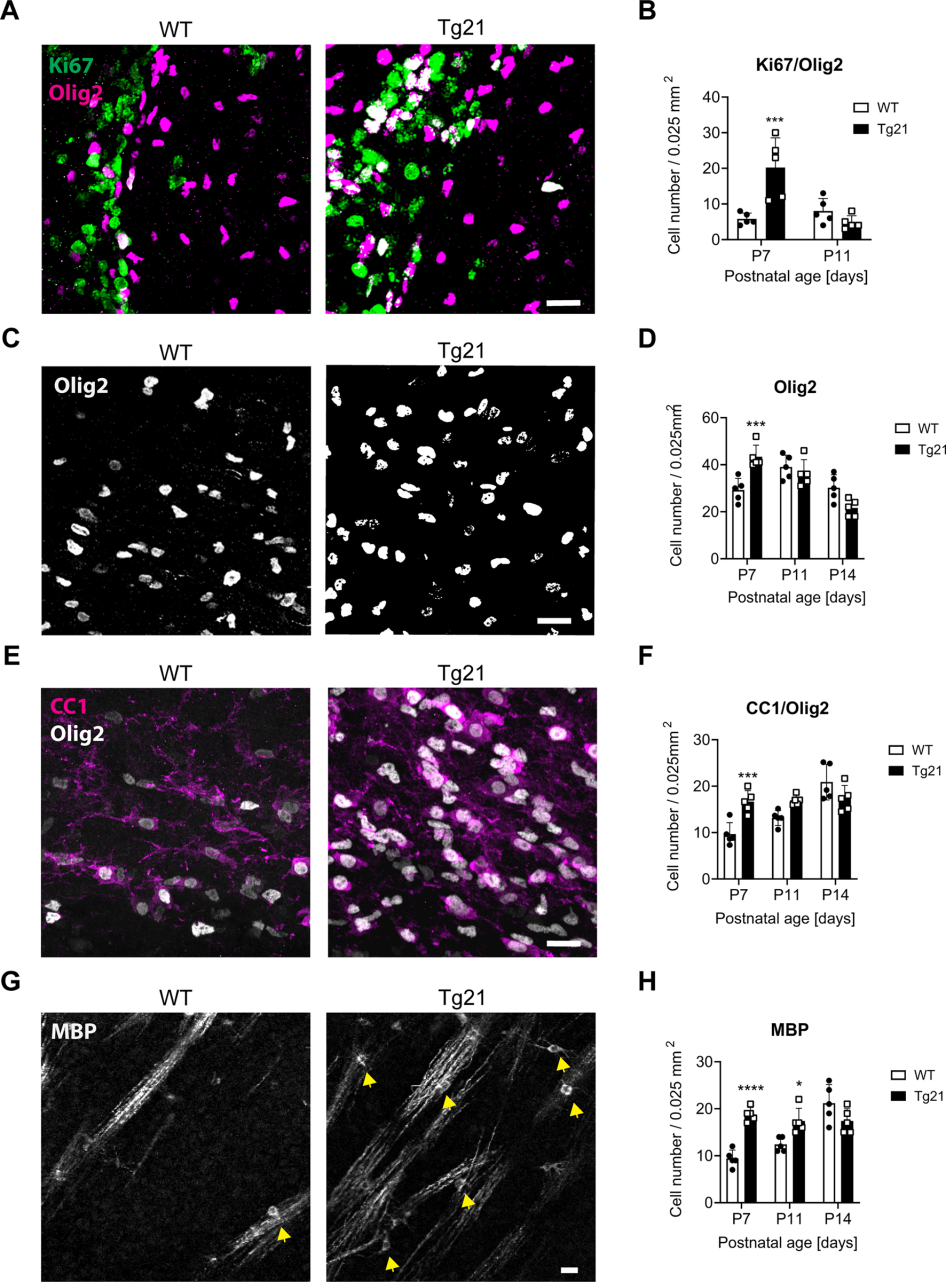
Enhanced OPC proliferation and maturation in Tg21 mice. (**A**) Representative confocal images of Ki67 (green) and Olig2 (magenta) immunofluorescence double staining in the SVZ at P7. Scale bar: 20 μm. (**B**) Analysis of the number of positive colocalized Ki67+/Olig2+ cells in the subventricular zone (SVZ) at P7 and P11 shows a significant increase in proliferation in the SVZ in the Tg21 mice at P7. 2-way ANOVA test, F(1,16) = 7.079, P = 0.0171. Multiple comparisons, ***P = 0.0009. N = 5 animals per genotype. (**C**) Representative confocal images of Olig2 immunofluorescence staining in the corpus callosum (CC) at P7. Scale bar: 20 μm. (**D**) Analysis of the number of Olig2+ cells in the CC at P7, P11, and P14, shows a significant increase at P7 in Tg21 mice. 2-way ANOVA, F(2,24) = 16.43, P < 0.0001. Multiple comparisons, ***P = 0.0005. N = 5 animals per genotype and age. (**E**) Representative confocal images of CC1 (magenta) and Olig2 (white) immunofluorescence double staining in the CC at P7. Scale bar: 20 μm. (**F**) Analysis of the number of CC1+/Olig2+ cells at P7, P11, and P14, showing a significant increase in Tg21 at P7. 2-way ANOVA, F(1,24) = 6.87, *P = 0.015. Multiple comparisons, ***P = 0.0006. N = 5 animals per genotype and age. (**G**) Representative confocal images of MBP immunofluorescence staining in the striatum (STR) at P7. Scale bar: 20 μm. (**H**) Analysis of the number of positive MBP cells (yellow arrows) in the STR at P7, P11, and P14, showing a significant increase at P7 and P11. 2-way ANOVA, F(1,24) = 14.94, ***P = 0.0007. Multiple comparisons, ****P < 0.0001, *P = 0.0127. N = 5 animals per genotype and age.

**Figure 6:**
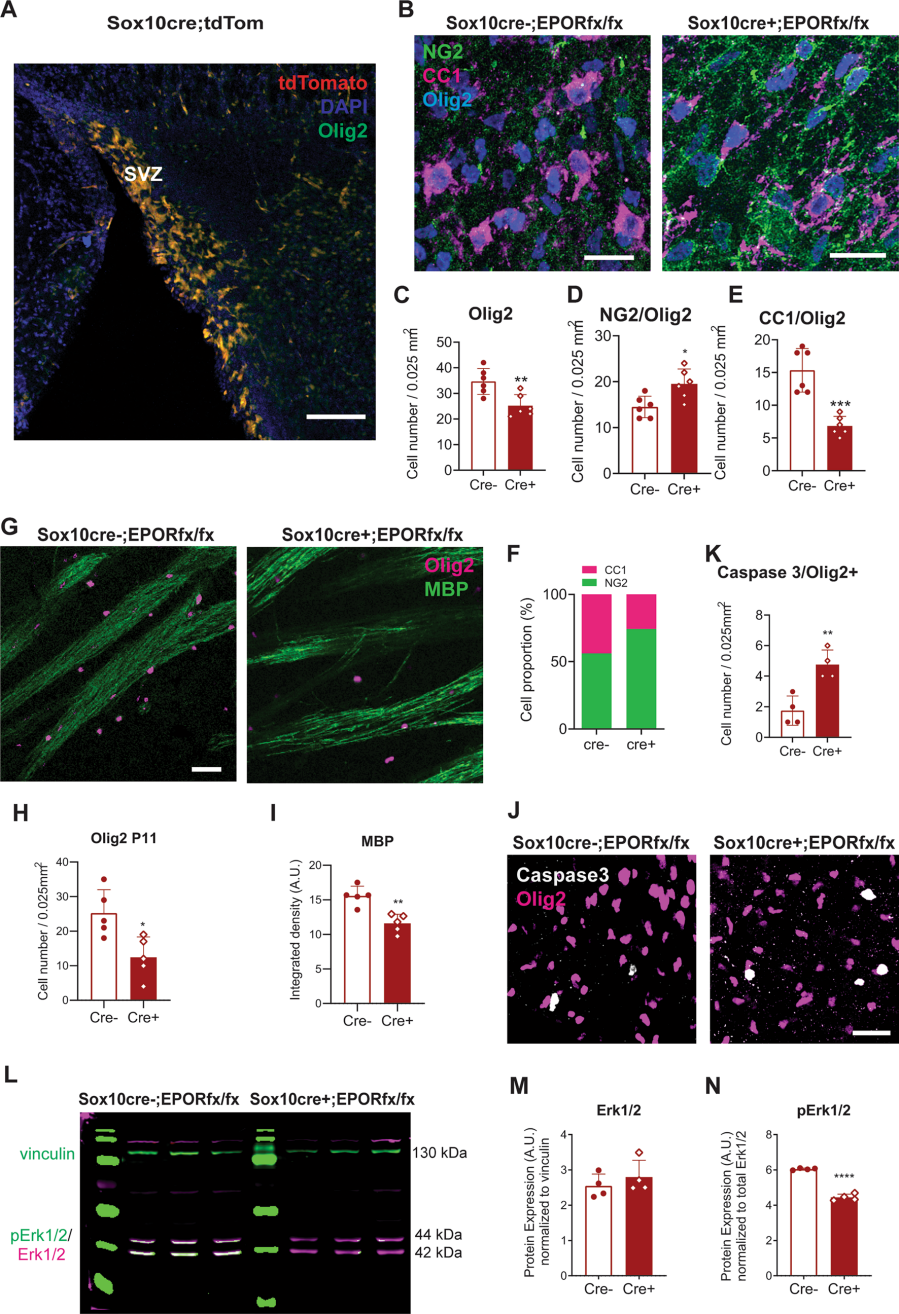
Removal of EPO receptors from OPCs causes a reduction in the number of mature oligodendrocytes and myelination. (**A**) Representative image of the SVZ zone in tissue from *Sox10*-tdTomato reporter line mouse at P7, showing the high expression of *Sox10* (red) cells in colocalization with Olig2 in this area. Scale bar 200 μm. (**B**) Representative confocal images of NG2, CC1, and Olig2 immunofluorescence triple staining at P7 in the STR of *Sox10-cre;EpoR*^*fx/fx*^ animals. Scale bar: 20 μm. (**C**) Analysis of the total Olig2+ cell number in *Sox10-cre;EpoR*^*fx/fx*^ mice at P7. Unpaired t-test: **P = 0.0058. N = 5 animals per genotype. (**D**) Analysis of NG2+ cell numbers in Cre+ mice at P7. Unpaired t-test: *P = 0.05, N = 5 animals per genotype. (**E**) Analysis of the total CC1+ cell number in *Sox10-cre;EpoR*^*fx/fx*^ mice at P7. Unpaired t-test: ***P = 0.0002. N = 5 animals per genotype. (**F**) NG2+/CC1 cells ratio in *Sox10-cre;EpoR*^*fx/fx*^ mice shows a 44% vs 29% OL maturation between Cre− and Cre+ mice. Unpaired t-test: ***P < 0.0001. (**G**) Representative confocal images of MBP and Olig2 immunofluorescence double staining at P7 in the STR of *Sox10-cre;EpoR*^*fx/fx*^ animals. Scale bar: 40 μm. (**H**) Analysis of the total Olig2+ cell number in *Sox10-cre;EpoR*^*fx/fx*^ mice at P11. Unpaired t-test: *P = 0.013. N = 5 animals per genotype. (**I**) Densitometric analysis of MBP expression in the striatum of *Sox10-cre;EpoR*^*fx/fx*^ mice at postnatal age P11. Unpaired t-test: **P = 0.0017. N = 5 animals per genotype. (**J**) Representative confocal images of caspase 3, and Olig2 double immunofluorescence staining in the SVZ at P4 of *Sox10-cre;EpoR*^*fx/fx*^ animals. Scale bar: 20 μm. (**K**) Analysis of Caspase3+/Olig2+ cells in *Sox10-cre;EpoR*^*fx/fx*^ mice at P4. Data are given as mean ± SD; Unpaired t-test: **P = 0.0044. N = 4 animals per age and genotype. (**L**) Western blot representative images of total Erk1/2 and phosphorylated Erk1/2 protein and vinculin as the loading control at P4 in *Sox10-cre;EpoR*^*fx/fx*^ mice. (**M**) Quantification of total Erk1/2 protein. Unpaired t-test. P = 0.24. N = 4 animals per genotype. (**N**) Quantification of phosphorylated (p-) Erk1/2 protein expression in cre+ and cre− mice at P4. Unpaired t-test, ****P < 0.0001. N = 4 animals per genotype.

After reporting EPO’s transient function in developmental myelination, we proceeded to evaluate the role of EPO in the postnatal maturation of the oligodendroglial lineage in the SVZ. We previously evaluated the postnatal expression of EPORs (Fig. 4), followed by an analysis of OL maturation in EPO-overexpressing mice (Fig. 5), and in mice with EPORs target deletion from the OL lineage (*Sox10*) (*Sox10-cre;EpoR*^*fx/fx*^* mice*) (Fig. 6).

Analysis of EPORs expression was performed in our two studied mouse models with fluorescent in situ hybridization (RNA Scope) of *Epor* mRNA in the SVZ and in OL transcription factor 2 expressing cells (Olig2 +), which is expressed throughout the entire OL lineage^35^. Brains of WT and Tg21 mice were evaluated at the postnatal ages P3, P7, P11, and P21 (Fig. 4A,B). We observed in WT and Tg21 genotypes that *Epor* expression in the SVZ of the third ventricle peaks at postnatal days P7 and P11 (Fig. 4A,B, the 2-way ANOVA, F(3,24) = 19.7, *P* < 0.0001). *Epor* expression, at P7, is two-fold higher in Tg21 mice than in WT mice (Fig. 4A,B, the 2-way ANOVA, F(1,24) = 11.2, *P* = 0.0027). Furthermore, combining FISH and immunofluorescent labelling of the Olig2+, at P7, *Epors* expression in Tg21 mice increased in Olig2+ cells (Fig. 4C, unpaired t-test, *P* = 0.0070, df = 24, and Fig. 4D), indicating a period for EPO signaling in developing OLs.

Next, we assessed *Epor* expression in *Sox10-cre;EpoR*^*fx/fx*^* mice* animals at P7 (Fig. 4E–H). Cre+ animals showed reduced *Epor* expression in the SVZ (Fig. 4E), cerebellum, and striatum, but not in the cerebral cortex and hippocampus (Fig. 4G, the 2-way ANOVA, F(1,30) = 23.64, P < 0.0001). In Cre+ animals, a reduction of *Epor* expression was quantified in Olig2+ cells in the SVZ (Fig. 4F,H, unpaired t-test, *P* = 0.0017, df = 30). Thus, the conditional deletion of *Epor* in the OL lineage was confirmed by the reduction in expression levels and allowed us to assess the impact of EPO signaling on OL maturation.

EPO regulation of postnatal oligodendrogenesis was morphologically analyzed in both our genetic mice lines in Olig2+ cells. OL proliferation was analyzed in the WT and Tg21 mice in the SVZ by double immunostaining Olig2 and the proliferation marker Ki67 at P7 and P11 (Fig. 5A,B). A higher number of total proliferating OL cells was quantified at P7 (Fig. 5B, the 2-way ANOVA, F(1,16) = 7.079, *P* = 0.0171); thus, constitutive EPO overexpression causes increased OL proliferation early in postnatal development. OPC originating in the SVZ migrate into the corpus callosum (CC), striatum and fornix to differentiate into mature myelinating oligodendrocytes^36^. Because the third wave of OPC maturation begins postnatally at day 4 (P4) after generation at P0^37^, and peaks at P15–21 in the CC^38^, we evaluated OL maturation in the CC at postnatal ages P7, P11, and P14 (Fig. 5C–H). Quantification of Olig2+ cells showed a significant genotype effect at P7 (Fig. 5C,D, the 2-way ANOVA, F(2,24) = 16.43, *P* < 0.0001). OL maturation was analyzed by double staining of Olig2, together with the mature lineage-specific marker (CC1)^39^. An increase in CC1+ cells was observed in the Tg21 mice at P7 (Fig. 5E,F, the 2-way ANOVA, F(1,24) = 6.867, *P* = 0.015). The increase in mature OLs caused by EPO in the CC could have resulted from a positive cell intrinsic drive to maturation, or an increase in survival. Finally, we assessed the number of MBP-expressing cells in the striatum, an area in which axons run less dense and in parallel, allowing them to be easily distinguished for MBP analysis. We observed an increased number at P7 and P11, showing that EPO increases the number of mature myelinating OLs (Fig. 5G,H, the 2-way ANOVA, F(1,24) = 14.94, *P* = 0.0007).

Next, the effect of EPORs deletion from the OL lineage (*Sox10*) on postnatal OL maturation was analyzed (Fig. 6). To confirm that the Cre-mediated deletion of EPOR in *Sox10*+ cells was specific to OLs, a *Sox10-*tdTomato reporter mouse line was first used. In this mouse line, tdTom expression was restricted to OLs (Olig2+ cells) and abundantly present at P7 in the SVZ (Fig. 6A). *Sox10-*tdTom cells were also distributed in the striatum partially colocalized with Olig2+ cells (Supplementary Fig. S1B); and a scattered distribution was found in the striatum and cortex colocalized with vascular pericytes (CD13)^40^ derived from the neural crest^41^ (Supplementary Fig. S1C). Because Sox10-tdTom+ cells colocalize primarily with Olig2+ cells, our work was specifically aimed at assessing the maturation of Olig2 lineage OPCs. Triple immunofluorescence staining for Olig2, together with OPC marker glycoprotein NG2^42^ and mature OL-specific marker (CC1) at P7 (Fig. 6B–F), showed a reduction in the total expression of Olig2+ cells in the Cre+ samples (Fig. 6C, Unpaired t-test, *P* = 0.0058, df = 10). While CC1+ mature OLs were also significantly reduced (Fig. 6E, Unpaired t-test, *P* = 0.0002, df = 10), a higher number of NG2+ cells were quantified in Cre+ mice (Fig. 6D, Unpaired t-test, *P* = 0.055), suggesting an effect of EPOR inactivation in OL maturation and consequently a proportionally higher number of immature OLs (Fig. 6F, the 2-way ANOVA, F(1,20) = 221.3, *P* < 0.0001). We further investigated myelination and total Olig2+ cell number in the striatum at postnatal age P11 by double immunofluorescence staining for Olig2 and MBP (Fig. 6G–I). At P11, a reduction in Olig2+ cell number (Fig. 6G,H, unpaired t-test, *P* = 0.013, df = 8) and MBP expression (Fig. 6G–I, Unpaired t-test, *P* = 0.0017, df = 8) was observed in Cre+ mice as compared to Cre− mice, suggesting a reduction in mature myelinating OLs. Given that EPO is an anti-apoptotic cytokine^12^ and that we previously showed that it promotes survival in hippocampal neurons during postnatal development^28^, we analyzed apoptosis (cleaved caspase 3) of Olig2+ cells of Cre+ and Cre− mice early at postnatal age P4. We observed increased OL apoptosis (cleaved caspase3+/Olig2+ cells) in Cre+ mice (Fig. 6J,K, unpaired t-test, *P* = 0.0044, df = 6). Thus, the lack of EPORs in immature OLs at an early stage of postnatal development increases apoptosis, causing a reduction in total Olig2+ cell numbers being a second possibility for the reduced number in CC1 cells.

We previously showed that EPO/EPOR cellular signaling acts in the developing brain through the Erk1/2 and AKT pathways^29^. Sustained Erk1/2-MAPK activation in OLs and Schwann cells is an important signal in promoting OL differentiation and developmental myelination^43–45^. Therefore, we evaluated the impact of EPOR loss from OPCs on total Erk1/2 protein expression along with phosphorylated (p) Erk1/2 expression at P4. Fluorescent western blot analyses of p-Erk1/2 (green) was normalized over total Erk1/2 (magenta) (Fig. 6L and Supplementary Fig. S2). The expression of total Erk1/2 was equal in Cre+ and Cre− mice (Fig. 6M, unpaired t-test, *P* = 0.24, df = 6), but p-Erk1/2 expression was decreased in Cre+ mice compared to Cre− (Fig. 6N, unpaired t-test, *P* < 0.0001, df = 6). Thus, the reduction of EPORs from OPCs reduces activation of the Erk1/2 pathway.

### EPO increases key pro-differentiating, pro-myelinating, and *Nfatc2/calcineurin* pathway transcripts in postnatal OPCs

We evaluated the expression of major pro-differentiating and pro-myelinating transcription factors in our two mice models. So far, most transcripts reported to be stimulated by EPO in OLs (mostly in injury) involve *PDGFαR* and *MBP*^16,46^. Here, we evaluated the expression of several transcription factors (see Table 3) identified as major determinants of embryonic and adult OL differentiation and myelination in our two animal models^47^. We also evaluated the expression of transcription factors from the Nfat/calcineurin signaling, an activity-dependent regulator of OPC differentiation and myelination in rodents and humans^48^.

**Figure 7:**
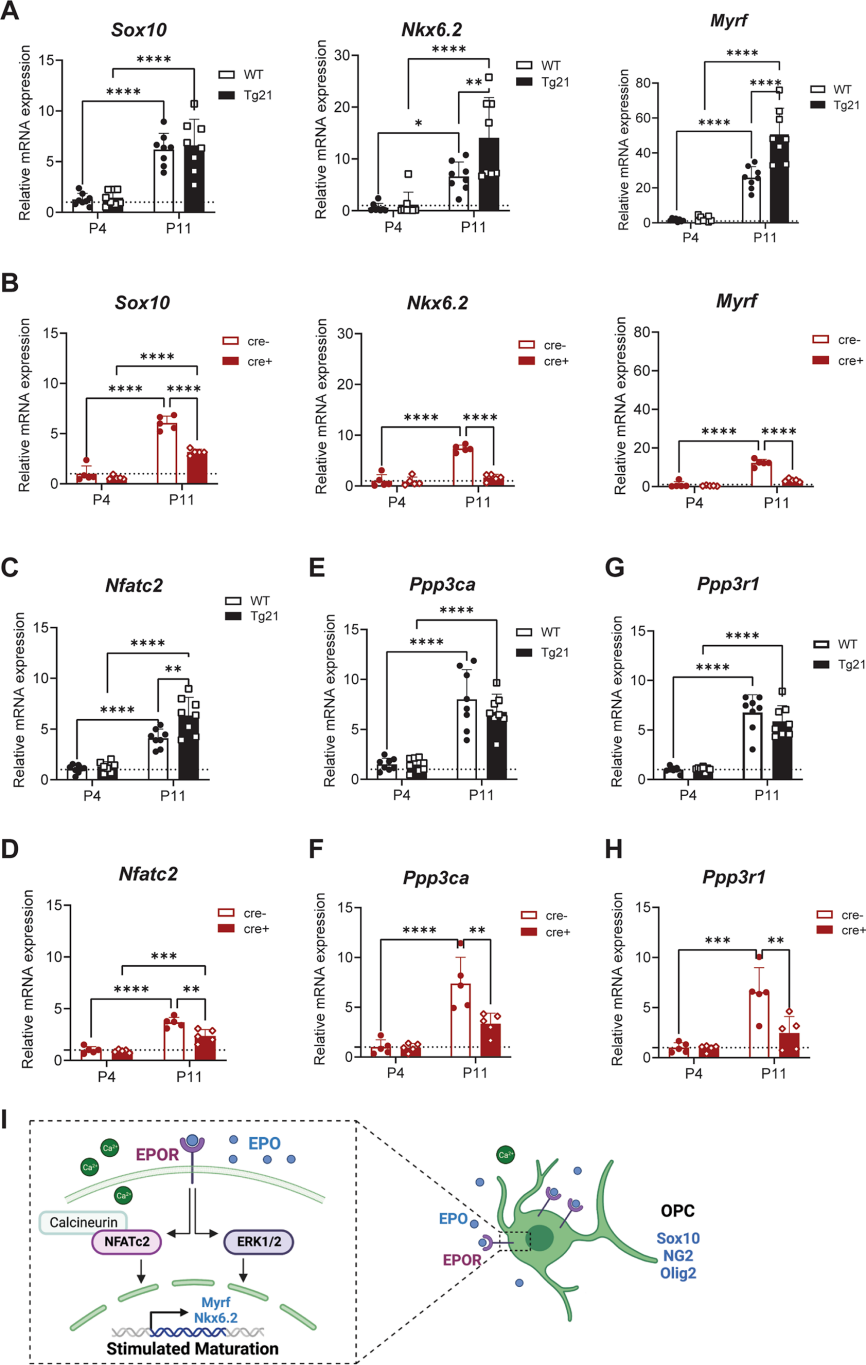
EPO increases key pro-differentiating, pro-myelinating, and *Nfatc2/calcineurin* pathway transcripts in postnatal OPCs. (**A**) *Sox10, Nkx6.2,* and *Myrf* mRNA expression in SVZ from WT and Tg21 mice at P4 and P11. *Sox10* expression (age: 2-way ANOVA, F(1,28) = 80.78, *P* < 0.0001). *Nkx6.2* expression, (age: 2-way ANOVA, F(1,28) = 38.14, *P* < 0.0001; genotype: 2-way ANOVA, F(1,28) = 6.83, *P* = 0.0143), and *Myrf* expression (age: 2-way ANOVA, F(1,28) = 158.5, *P* < 0.0001; genotype: 2-way ANOVA, F(1,28) = 19.4, *P* = 0.0001). Multiple comparisons: ****P < 0.0001, *P < 0.05, **P < 0.01. N = 8 animals per age and genotype. (**B**) *Sox10, Nkx6.2,* and *Myrf* mRNA expression in SVZ from *Sox10-cre;EpoR*^*fx/fx*^ mice at P4 and P11. *Sox10* expression (age: 2-way ANOVA, F(1,16) = 242.7, *P* < 0.0001; genotype: 2-way ANOVA:, F(1,16) = 242.7, *P* < 0.0001); *Nkx6.2*, (genotype: 2-way ANOVA, F(1,16) = 79.56, *P* < 0.000); *Myrf* (genotype: 2-way ANOVA, F(1,16) = 155.3, *P* < 0.0001). Multiple comparisons: ****P < 0.0001. N = 5 animals per age and genotype. (**C**) *Nfatc2* mRNA expression in SVZ from WT and Tg21 mice at P4 and P11. (age: 2-way ANOVA. F(1,28) = 119.3, *P* < 0.0001; genotype: 2-way ANOVA, F(1,28) = 11.8, *P* = 0.0024). Multiple comparisons: **P = 0.0011, ****P < 0.0001. N = 8 animals per age and genotype. (**D**) *Nfatc2* mRNA expression in SVZ from *Sox10-cre;EpoR*^*fx/fx*^ mice at P4 and P11. (age: 2-way ANOVA, F(1,16) = 114.7, *P* < 0.0001; genotype: 2-way ANOVA, F(1,16) = 13.46, *P* = 0.0021). Multiple comparisons: **P = 0.01, ***P = 0.0004, ****P < 0.0001. N = 5 animals per age and genotype. (**E**) *Ppp3ca* mRNA expression in SVZ from WT and Tg21 mice at P4 and P11. (Age: 2-way ANOVA, F(1,28) = 86.33, *P* < 0.0001). Multiple comparisons: ****P < 0.0001. N = 8 animals per age and genotype. (**F**) *Ppp3ca* mRNA expression in SVZ from *Sox10-cre;EpoR*^*fx/fx*^ mice at P4 and P11. (Genotype: 2-way ANOVA, F(1,16) = 9.56, **P = 0.0077). Multiple comparisons: **P = 0.0011, ****P < 0.0001. N = 5 animals per age and genotype. (**G**) *Ppp3r1* mRNA expression in SVZ from WT and Tg21 mice. (age: 2-way ANOVA, F(1,28) = 153.1, *P* < 0.0001). Multiple comparisons: ****P < 0.0001. N = 8 animals per age and genotype. (**H**) *Ppp3r1* mRNA expression in SVZ from *Sox10-cre;EpoR*^*fx/fx*^ mice at P4 and P11. (Age: 2-way ANOVA, F(1,16) = 27.57, *P* = 0.0001; genotype: 2-way ANOVA, F(1,16) = 9.45, *P* = 0.0073). Multiple comparisons: **P = 0.0028, ***P < 0.001. N = 5 animals per age and genotype. (**I**) Suggested mechanism of EPO action in OPC from the SVZ during postnatal development.

The evaluation was done by RT-PCR analysis in tissue containing the SVZ at P4 and P11 (Fig. 7). An age-dependent increase in expression of *Sox10* (4.7-fold, 2-way ANOVA, F(1,28) = 80.78, *P* < 0.0001)*, Nkx6.2* (11.8-fold, 2-way ANOVA, F(1,28) = 38.14, *P* < 0.0001), and *Myrf* (20.3-fold, 2-way ANOVA, F(1,28) = 158.5, *P* < 0.0001) was observed in WT and Tg21 mice (Fig. 7A). At P11, Tg21 mice showed higher expression in *Nkx6.2* (2.1-fold, 2-way ANOVA, F(1,28) = 6.83, *P* = 0.0143), and *Myrf* (2.0-fold, 2-way ANOVA, F(1,28) = 19.4, *P* = 0.0001) than WT control (Fig. 7A). In the *Sox10-cre*^*Tg/*+^*;EpoR*^*fx/fx*^ (Cre +) mice, less age-dependent increase in the expression of *Sox10* (0.27-fold, 2-way ANOVA, F(1,16) = 73.32, *P* < 0.0001), *Nkx6.2* (4.8-fold, 2-way ANOVA, F(1,16) = 79.56, P < 0.0001), and *Myrf* (10.8-fold, 2-way ANOVA, F(1,16) = 155.3, *P* < 0.0001) was observed than in control (cre-) (Fig. 7B).

We next evaluated the impact of EPO in Nfatc (*Nfatc1-4*), calcineurin A (*Ppp3ca, Ppp3cb, Ppp3cc*), and calcineurin B (*Ppp3r1* and *Ppp3r2*) transcription expression. An age-dependent increase, mainly in the expression of *Nfatc2* (4.5-fold, 2-way ANOVA, F(1,28) = 119.3, *P* < 0.0001), was observed in WT and Tg21 mice (Fig. 7C). Moreover, at P11, Tg21 mice showed higher expression in *Nfatc2* (1.6-fold, 2-way ANOVA, F(1,28) = 11.8, *P* = 0.0024) than WT (Fig. 7C). Also, an age-dependent increase in expression of *Nfatc2* was observed in *Sox10-cre;EpoR*^*fx/fx*^ cre+ mice and control samples (3.2-fold, 2-way ANOVA, F(1,16) = 114.7, *P* < 0.0001), but the expression increase was lower in cre+ mice (0.69-fold, 2-way ANOVA, F(1,16) = 13.46, *P* = 0.0021) (Fig. 7D). Thus, our results indicate that EPO signaling onto postnatal OPCs increases *Nfatc2* expression*.*

Analysis of calcineurin A isoforms showed an age-dependent increase mainly in *Ppp3ca* in WT and Tg21 mice (fivefold, 2-way ANOVA, F(1,28) = 86.33, *P* < 0.0001, Fig. 7E). In *Sox10-cre;EpoR*^*fx/fx*^ mice, an age- (5.5-fold, 2-way ANOVA, F(1,16) = 43.8, *P* < 0.0001) and a genotype-dependent increase was observed in the *Ppp3ca*, with a lower expression increase in Cre+ mice samples (0.53-fold, 2-way ANOVA, F(1,16) = 9.56, *P* = 0.0077, Fig. 7F). Finally, analysis of calcineurin B isoforms showed only expression of the *Ppp3r1* isoform in all genotype samples (Fig. 7G,H). In Tg21 mice, an age-dependent increase was observed (6.1-fold, 2-way ANOVA, F(1,28) = 153.1, *P* < 0.0001, Fig. 7G). Moreover, in *Sox10-cre;EpoR*^*fx/fx*^ mice an age (4.6-fold, 2-way ANOVA, F(1,16) = 27.57, *P* = 0.0001, Fig. 7H) and genotype-dependent increase in *Ppp3r1* expression was observed, with a lower increase in Cre+ mice samples at P11 (0.45-fold, 2-way ANOVA, F(1,16) = 9.45, *P* = 0.0073), Fig. 7H). Our data show that EPO signaling onto OPC enhances calcineurin A *Ppp3ca* and calcineurin B *Ppp3r1* isoforms expression*.* In summary, we show an increase in *Nfatc2, Ppp3ca and Ppp3r1* transcription factors suggesting that beside activating EPO the Erk1/2 pathway, may also activate the Nfatc2/calcineurin pathway in OLs (Fig. 7I).

### EPO stimulates postnatal motoric development, coordination, and learning

**Figure 8:**
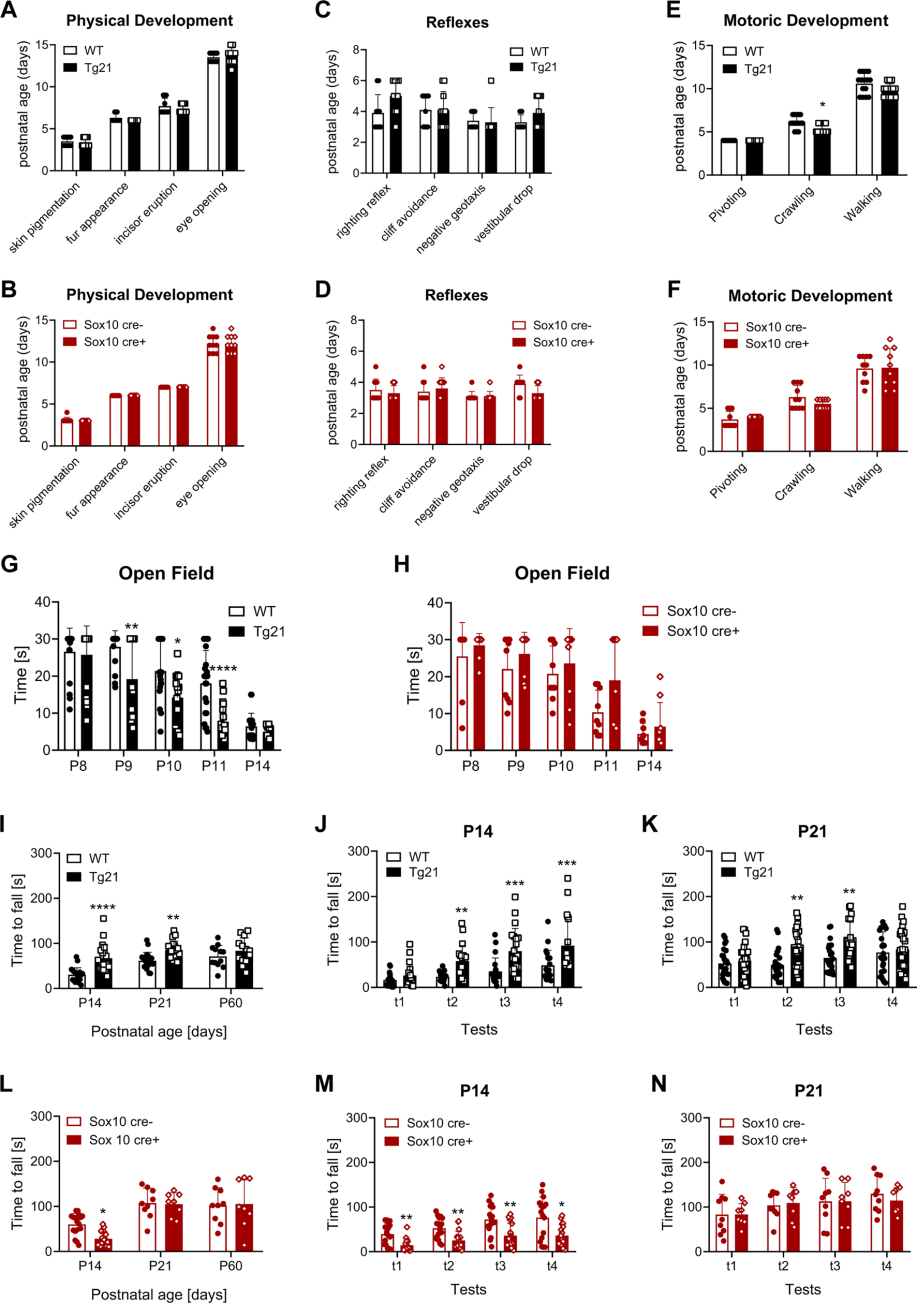
EPO stimulates motoric development and motor coordination learning. Physical development in: (**A**) WT and Tg21 mice, and (**B**) *Sox10-cre;EpoR*^*fx/fx*^ mice**,** showing the postnatal age at which skin pigmentation, fur appearance, incisor eruption, and eye-opening occurred. N = 10 animals per genotype. Appearance of reflexes in: (**C**) WT and Tg21 mice, and (**D**) *Sox10-cre;E10-poR*^*fx/fx*^ mice. Shown are righting reflexes, cliff avoidance, negative geotaxis, and vestibular drop. N = 10 animals per genotype. Motoric development in: (**E**) WT and Tg21 mice, N = 15 animals per genotype; 2-way ANOVA, F(2,56) = 3.18, *P = 0.048, and (**F**) *Sox10-cre;EpoR*^*fx/fx*^ mice. N = 10 animals per genotype. Shown are the ages at which pups started pivoting, crawling, and walking. Motoric open field test across postnatal ages P8 to P14 in: (**G**) WT and Tg21 mice. 2-way ANOVA, F(1,169) = 30.98, ****P < 0.0001). Multiple comparisons: *P = 0.0134, **P = 0.0015, ****P < 0.0001, N = 12–20 animals per genotype and age, and (**H**) *Sox10-cre;EpoR*^*fx/fx*^ mice. N = 6–10 animals per genotype and age. (**I**–**K**) Rotarod motor coordination tests across postnatal ages in WT and Tg21 mice. (**I**) Shown are the average values from the four trials per animal at P14, P21 and P60. 2-way ANOVA, F(1,114) = 27.48, ****P < 0.0001. Multiple comparison: **P = 0.0051, ****P < 0.0001. N = 20 animals per genotype and age. (**J**) Rotarod motor coordination tests in WT and Tg21 mice at P14. Shown are all four trials per test. 2-way ANOVA, F(1,152) = 35.36, *P* < 0.0001. Multiple comparisons: **P = 0.0093, ***P = 0.0006. N = 20 animals per genotype (**K**) Rotarod motor coordination tests in WT and Tg21 mice at P21. Shown are all four trials per test. 2-way ANOVA, F(1,152) = 9.561, *P* = 0.0034. Multiple comparisons: **P = 0.0052. N = 19 animals per genotype. (**L**–**N**) Rotarod motor coordination tests across postnatal ages in *Sox10-cre;EpoR*^*fx/fx*^ mice. (**L**) Average values from the four trials per animal at P14, P21 and P60. N = 8–15 animals per genotype and age. (**M**) Rotarod motor coordination tests in *Sox10-cre;EpoR*^*fx/fx*^ mice at P14. Shown are all four trials per test. 2-way ANOVA, F(1,112) = 21.46, *P* < 0.0001. Multiple comparisons: *P = 0.0159, **P = 0.0055. N = 15 animals per genotype. (**N**) Rotarod motor coordination tests in *Sox10-cre;EpoR*^*fx/fx*^ mice at P21. Shown are all four trials per test. N = 9 cre− and 8 cre+ animals.

We performed behavioral experiments to evaluate how EPO brain overexpression and EPOR deletion from OPCs and Schwann cells (also regulated by *Sox10* and responsive to EPO in their maturation and myelination) influences postnatal motoric development. Postnatal physical development, reflexes, and motoric development were examined in Tg21 and *Sox10-cre;EpoR*^*fx/fx*^ mice lines to detect possible growth or physical development alterations. In both mouse lines, there was no difference from controls in physical development. Skin pigmentation (P3–P4), fur appearance (P6–P7), and incisor eruption (P7–P9) occurred at similar ages (Fig. 8A,B). The two lines slightly differed in their postnatal development in the eye-opening period (days). In the *Sox10-cre;EpoR*^*fx/fx*^ mouse line (mixed background), eye-opening occurred between days P11-P14, and in the Tg21 mice line (Bl6C57 background) between P12-P15. The onset of reflexes was not different between mutated animals and their controls (Fig. 8C,D).

Next, we tested the transition from pivoting to crawling and walking. In Tg21 and WT mice, pivoting was already established at P4, crawling started at P5, and was fully acquired by P7 in WT and P6 in Tg21 mice. Quadrupedal locomotion was first detected at P8 in WT and Tg21 pups and was normal in all WT pups at P12 and all Tg21 pups at P11. Therefore, complete motor development occurred one day earlier in Tg21 than control (Fig. 8E, the 2-way ANOVA, F(2,56) = 3.188, P = 0.048). In the *Sox10-cre;EpoR*^*fx/fx*^ mice, pivoting started at P3 and was already established at P5, and crawling started at P5 and was fully acquired by P8 in all pups. Quadrupedal locomotion was first detected at P7, was normal at P11 in all Cre− pups, but was normal in all Cre+ pups only at P13. Thus, some Cre+ mice animals showed two days delayed in completing their motor development (Fig. 8F). Together, our data suggest that EPO CNS overexpression stimulates the development of the motor system.

After animals were able to walk, locomotion speed was assessed in a motoric open field test, measuring the time (maximal 30 s given time) needed to exit a circle in the center of an arena, performed between P8 and P14. A larger fraction of Tg21 mice than WT mice was able to perform the test, and the average time needed to exit the circle was shorter in Tg21 mice, reaching statistical significance at P9, P10, and P11 (Fig. 8G, the 2-way ANOVA, F(1,169) = 30.98, *P* < 0.0001). In *Sox10-cre;EpoR*^*fx/fx*^ mice, a larger fraction of Cre+ animals were unable to perform the open field test (Fig. 8H, the 2-way ANOVA, F(1,80) = 6.77, *P* = 0.011). Thus, our data further indicate that EPO brain overexpression stimulates motor coordination but EPOR deletion from the *Sox10* line delays it.

The effects of EPO on motor coordination were further tested with a rotarod apparatus at P14, P21, and P60. The results showed a significant effect of genotype and age, with Tg21 mice having a longer latency to fall (better performance) at P14 and P21 than WT (Fig. 8I, the 2-way ANOVA, F(1,114) = 27.48, P < 0.0001). At P14, the difference was mainly apparent during the late trials, suggesting better learning performance by the mutant mice (Fig. 8J, the 2-way ANOVA; F(1,152) = 35.36, *P* < 0.0001). Likewise, motor learning was better in Tg21 mice at P21, reaching a ceiling effect in the third trial (Fig. 8K, the 2-way ANOVA; F(1,152) = 9.561, *P* = 0.0034). Therefore, EPO overexpression stimulates postnatal motor coordination and motor learning. The effects of EPOR deletion from the *Sox10-cre* line showed an impact at P14, with Cre+ pups unable to perform the test (Fig. 8L, the 2-way ANOVA, F(1,62) = 1.776, P = 0.1875). At P14, in Cre+ pups, the motor coordination learning process across trials was impaired (Fig. 8M, the 2-way ANOVA; F(1,112) = 21.46, *P* < 0.0001). At P21, motor learning was equal between Cre+ and Cre− pups (Fig. 8N, the 2-way ANOVA; F(1,60) = 0.02738, *P* = 0.8708). Thus, a transient motor coordination and learning impairment was observed in *Sox10-cre;EpoR*^*fx/fx*^ mice. This might reflect the delay in the maturation of myelinating OLs and Schwann cells. Together, our behavioral data suggest that EPO/EPOR signaling stimulates motoric development and motor coordination learning.

## Discussion

This study provides morphological, ultrastructural, molecular, and behavioral evidence for the crucial role of EPO in the normal (physiological) stimulation of developmental myelination in the mouse brain and improvement in motoric coordination learning. We show that EPO increases the proliferation of Olig2+ cells, prevents apoptosis, and stimulates maturation towards fully mature myelinating OLs. We show that *Epors* are highly expressed during the first two postnatal weeks in the SVZ and that EPO signals on postnatal OPCs activates the Erk1/2 pathway and the transcription from the Nfatc2 pathway and essential oligodendrocyte pro-differentiation and pro-maturation factors.

We show that constitutive EPO overexpression in the brain leads to a higher density of axons, an earlier onset of the myelination process, visible at P7, and a faster rate of axonal ensheathment, as seen morphologically and ultrastructurally. Notably, axons were myelinated independent of their diameter, while the number and consequent thickness of myelin sheaths were higher in Tg21 mice at an earlier stage in development. Thus, EPO increases the speed but not the extent of developmental myelination. We previously showed that EPO overexpression increases postnatal synaptic density and accelerates neuronal maturation^28,29^, meaning that the earlier synaptogenesis and circuit maturation stimulated by EPO is accompanied by a faster myelination process and brain growth that reaches normality at earlier adulthood. Indeed, improved GABAergic development can act as a local environment cue to control myelination and OL lineage cell number^49^. The impact of EPO overexpression speeding up the process of brain myelination without alterations in adulthood suggests the potential for EPO administration in treating brain developmental hypomyelination. Indeed, systemic administration of EPO for 3 weeks in young adult mice at sufficiently high concentrations to cross the blood–brain barrier has been shown to increase myelin proteins^18^.

EPORs are expressed in the developing mouse brain mid-gestation, localized in the neural tube and the neuroepithelium containing proliferating neuro-precursors^50,51^. Here we report a high level of *Epor* expression in OPC (Olig2+/*Sox10*+) in the SVZ and striatum at postnatal days P7 and P11, which markedly decreases in expression towards adulthood. At P7 an increase in Olig2+ cell proliferation is observed in our Tg21 mice. EPO has also been shown to stimulate OPC proliferation and differentiation in the SVZ in response to various types of insult, including prenatal ischemia^16^ and demyelinating diseases^52,53^. However, treatments of EPO in juvenile mice (P28), showed no impact on the proliferation of hippocampal parenchymal OPCs^18^. EPOR expression in the hippocampus is not as high as in the SVZ area during early postnatal ages. Moreover, parenchymal OPCs respond differently than SVZ OPCs to growth factors such as (PDGF)^54^. Consequently, our model allows early postnatal stimulation of OPC proliferation and differentiation in the SVZ. A transient significantly increased expression of mature OLs (CC1+) and total Olig2+ cells was observed at P7 and P11 in Tg21 mice, indicating that EPO accelerates the process of oligodendrocyte maturation. Inversely, in the *Sox10-cre;EpoR*^*fx/fx*^ mice line, SVZ OPC postnatal maturation in the Cre+ mice was delayed, reflected by a reduced number of CC1+ cells at P7. This reduction in mature CC1 was accompanied by reduced *Epor* expression in Olig2+ cells and increased apoptosis in the SVZ, the striatum, and the cerebellum. Analysis of local proliferation of Olig2+ cells showed an increase in proliferative OLs in Tg21 mice at P7. Thus, we suggest that EPO is proliferative and anti-apoptotic, and plays a physiological role in the maturation of OPCs, which adds a new function for EPO in postnatal brain maturation. The transient effects of EPO in development are regulated by the high expression levels of *Epors*. EPO’s stimulation on astrocytes and microglia was not addressed in this study, two cell types that may also contribute to myelination control. In addition, in the *Sox10-cre;EpoR*^*fx/fx*^ mice line EPORs from sparse neuronal crest-derived pericytes were also deleted in striatum and cortex, thus there may also exist potential interactions between pericytes and OPCs in perivascular regions^55^.

We have previously shown that mice overexpressing EPO in the brain activate the JAK2 Erk1/2 and AKT pathways postnatally^29^. In this study, we additionally elucidated those signaling pathways in the *Sox10-cre;EpoR*^*fx/fx*^ mice line and determined a reduction in phosphorylation of Erk1/2 in the Cre+ mice. Thus, EPO signaling in OPCs activates the Erk1/2-MAPK pathway, a signal that promotes differentiation in OLs and Schwann cells as well as developmental myelination^43–45^. Schwann cell development and peripheral myelination may also be affected in our loss of function mice model. Erk1/2 inhibition was shown to induce re-myelination in vitro and animal models of acute experimental autoimmune encephalomyelitis^56,57^, but not in chronic demyelination processes^57^. Erk1/2 signaling is also a central regulator of immune cell function^58^. Thus, the process of re-myelination is influenced by the immune response. Conversely, during developmental myelination, Erk1/2 activation is influenced by growth factors, such as EPO, that stimulate differentiation and maturation of OPCs. We measured the expression of several key pro-differentiating and pro-myelinating transcription factors to determine which are stimulated by EPO to induce OPC to OL maturation and myelination. We provide evidence that EPO enhances the expression of *Nkx6.2* and *Myrf*, both transcription factors involved in regulating OL maturation and myelination during postnatal stages^59,60^. *Nkx6.2* is selectively expressed in Olig2+ cells of OL lineage and upregulates as OPCs undergo terminal differentiation, suppressing the expression of *Nkx2.2,* which initially expresses in differentiating OPCs^61^. *Myrf* is induced during terminal OPC differentiation, suppressing essential genes in earlier phases of the oligodendroglial development^62^. The increased expression of *Nkx6.2* and *Myrf* transcription with EPO and the reduction in expression upon EPOR deletion from Sox+ cells indicate that EPO signaling induces postnatal terminal oligodendrocyte differentiation. We also provide the first evidence that EPO enhances the expression of *Nfatc2* and *calcineurin* transcripts in OPCs. *Nfatc2* is reportedly a highly expressed family member under the direct control of *Sox10* in rodent oligodendroglial cells and coincides with increased calcineurin activation^48^. Moreover, calcineurin is usually activated by an increase in intracellular calcium levels that occur at the onset of OPC differentiation^63^, mediated by voltage-gated calcium or calcium-permeable glutamate channels^64,65^. Our previous data, showing enhanced synaptogenesis, fits with the observed increase in the activity-dependent transcripts and underscores the clinical value of upregulating *Nfatc2* with EPO to stimulate oligodendroglial differentiation and remyelination. The observed increase in transcription factors with EPO and decrease without EPORs which could also result from the increased or decreased number in mature Olig2 cells. Thus, whether the EPO-stimulated Erk1/2 pathway controls the regulation of those transcripts remains to be determined.

Finally, we show that EPO overexpression in the CNS leads to improved postnatal motor coordination and learning and that deletion of EPORs from OPCs and Schwann cells causes motoric impairment at P14. Higher myelination rates in the central and peripheric nervous systems are associated with improved motoric coordination, whereas hypomyelination with poor motoric coordination, likely due to the functioning of the myelin sheath on the efficiency of synaptic signals traveling along axons and the maintenance of high conduction speeds. Therefore, EPO’s impact in postnatal motor coordination and motor learning most likely results from EPO stimulating developmental myelination in OLs and Schwann cells. Indeed, EPORs are expressed in Schwann cells after injury^66^, but expression during normal development requires further investigation.

In summary, our data fills an important gap in our knowledge about the physiological role of EPO in the developmental myelination process in the mouse brain. EPORs are developmentally regulated and stimulate differentiation of OPCs towards fully myelinating OLs. EPO accelerates developmental myelination and functionally improves motoric coordination and motoric learning without causing any alterations in total brain volume and myelination in adulthood. Considering that endogenous EPO production in the brain and EPORs expression are altered in preterm neonates^67–71^, our results explain, at least in part, the cause of impaired myelination in specific areas of the brain of preterm neonates. Similarly, our results strongly suggest that EPO is a promising pro-myelinating agent to overcome this impairment, although the efficiency may vary according to age and injury type and severity.

## Materials and methods

### Transgenic animals

The following constitutive transgenic lines were used in our study: T*gN(PDGFB-EPO)322ZbZ (Tg21)* overexpressing neuronal EPO in the CNS^26,27^; *Sox10-cre/J* (JAX #025807)^72^; *EPOR floxed (*kindly provided by Prof. Christian Grimm, University of Zurich, Zurich, Switzerland). *Sox10-Cre* animals were crossed with homozygous EPOR *floxed* mice to achieve Cre-mediated deletion of EPOR in cells expressing *Sox10*, namely OPC. *Sox10-Cre*^*Tg/*+^*/EpoR*^*fx/fx*^ (cre+) mice were compared to littermates *Sox10-Cre*^+*/*+^*/EpoR*^*fx/fx*^ (cre-) in the study.

Tg21 mice were bred on a C57BL/6 background at the Laboratory Animal Service Center of the University of Zurich, and *Sox10* and EPOR *flox* mice had a mixed background.

Animal experiments were approved by the Cantonal Veterinary Office of Zurich, Switzerland (license number 177/2016). Experiments were performed in accordance with the official guidelines and regulations, following the ARRIVE guidelines^73^. Animals were kept in standard housing conditions with food and water provided ad libitum*.* At least four male animals per genotype and age were collected from four different littermates and used for each experiment. The study concentrated on male animals due to their higher vulnerability to white matter injury.

### Tissue processing for H&E and Immunohistochemistry

Tissue from both transgenic lines and their controls was collected at postnatal days (P) P3, P7, P11, P14, P21, P25, and P60. For each specific experiment, different ages were taken, as specified in the results section. After anesthetization by i.p. overdose of pentobarbital (50 mg/kg i.p., Kantonsapotheke Zürich, CH, see Table 1 for volumes), mice were perfused with cold phosphate-buffered saline solution (PBS; pH 7.4), followed by paraformaldehyde (4%) (PFA) fixation. The brains were collected, sagittal cut along the brain midline, or kept intact for coronal cuts, and postfixed with 4% PFA at 4 °C for a time, varying according to age (see Table 1). Brains were then cryoprotected in 30% sucrose for 24 to 72 h at 4 °C until tissues sank and stored at − 20 °C. Brains were embedded in optimal cutting temperature (OCT) medium (VWR International) and cut in sagittal or coronal serial sections with a sliding microtome (MICROM HM 400, MICROM International GmbH, Walldorf, Germany). Thickness and series sample fraction (ssf) varied depending on mouse age (see Table 1). Tissues were stored at − 20 °C in an antifreeze solution until immunohistochemical staining.

**Table 1:** Perfusion and brain cutting thickness parameters.

Age group	Pentobarbital volume (µl)	Needle size (G)	Pump rate (rpm)	Postfixation time (h)	Cut thickness (µm)	Series sample fraction (µm)
P3	10	27	Manual	72	60	240
P7	20	26	0.4	24	50	300
P11	30	25	0.4	18	40	320
P14	40	25	0.5	12	40	400
P21	50	24	0.9	3	40	480
P25	100	24	1.2	3	40	480

### H&E staining

A series of tissues at ages P3, P7, P14, and P25 were mounted on gelatin-coated glass slides (Thermo Scientific, Menzel, GmbH) and allowed to air-dry overnight at room temperature (RT). Tissues were immersed in dH_2_O for 1 min, followed by immersion in hematoxylin solution (MHS16, Mayer, Sigma-Aldrich Chemie GmbH, Germany) for 30 s and rinsed in water for 1 min. The step was repeated depending on the desired intensity. Tissues were then immersed in 1% eosin (E4009, Sigma-Aldrich Chemie GmbH, Germany) solution for 20 s. Slides were dehydrated in ethanol of increasing concentrations (2 × 70%, 2 × 96%, 3 × 100%) for 5 min each, followed by clearing in xylene 4 times for 5 min. On the following day, the sections were coverslipped with Eukitt mounting medium (Fluka analytical, Sigma-Aldrich Chemie GmbH, Germany), let to dry overnight, and long-termed stored at RT.

### Immunoperoxidase staining

Myelinated areas in the brain were quantified in serial coronal sections at P3, P7, P11, P14, and P21 immunolabeled for myelin basic protein (MBP). Tissues were transferred from the antifreeze solution to a 12-well plastic plate filled with Tris-Triton (pH 7.4) for washing; tissues were then incubated with primary antibody overnight at 4 °C under constant agitation in a solution containing 0.2% Triton X-100 and 2% normal serum (NS). Following three washes, a biotinylated-conjugated antibody (goat anti-rabbit, Jackson Immunoresearch, 1:300) was applied and incubated for 30 min, at RT, in a moist chamber under continuous agitation. Brain tissues were then washed in Tris-Triton three times and incubated in Avidin–Biotin Complex (ABC) solution (Vectastain Elite ABC Kit standard, Vector Laboratories, Burlingame USA) under continuous agitation for 30 min at RT and rinsed again three times. Tissue sections were pre-incubated with 3,3’-diaminobenzidine tetrahydrochloride (DAB)-containing Tris-Triton with 0.05% Triton X-100 for 5 min under agitation; the reaction was then started by adding DAB solution containing 0.01% hydrogen peroxidase. After 5–7 min, depending on the intensity of the staining, the reaction was stopped by transferring the sections into ice-cold PBS, followed immediately by another three washing steps. The sections were mounted on gelatin-coated glass slides and left to dry overnight. On the following day, they were dehydrated, coverslipped with Eukitt mounting medium, and long-termed stored at RT.

### Immunofluorescence stains

Double or triple immunofluorescence staining at P7, P11, and/or P14 was used to analyze multiple markers within the same section. The tissues were double stained for analysis of myelinated axons (NF200 and MBP), OL proliferation (Ki67 and Olig2), myelinating OLs (MBP and Olig2), OL apoptosis (Caspase 3 and Olig2), and *Sox10-*tdTom cell expression specificity (tdTom and DAPI). The tissues were double (CC1 and Olig2) or triple stained for analysis of OL maturation (NG2, CC1, and Olig2). Free-floating sections were washed 3 times before incubating with the first antibodies (see Table 2) overnight at 4 °C under continuous agitation in a solution containing 2% Triton X-100 and 2% NGS. After washing the sections three times, they were incubated with the secondary antibodies raised in donkey against the different species of the primary antibodies and coupled to either Alexa488, Cy3, or Alexa647 in a solution containing DAPI and 2% NGS, at RT for 30 min in the dark. After another washing step of 3 times, sections were mounted on gelatin-coated glass slides, coverslipped with Dako fluorescence mounting medium (Dako, Carpinteria CA, USA), and stored in a closed cardboard box at 4 °C.

**Table 2:** Primary antibodies were used.

Target	Species	Used dilution	Company
Myelin Basic Protein (MBP)	Rabbit, polyclonal, IgG	1:500	Chemicon (AB5864)
Neurofilament 200 (Nf-200)	Mouse, monoclonal, IgG	1:1000	Sigma (N5389)
Olig2	Goat, polyclonal, IgG	1:500	R&D Systems (AF2418)
Ki67	Rabbit polyclonal, IgG	1:300	Abcam (15580)
NG2	Rabbit, monoclonal, IgG	1:1000	Abcam (ab275024)
CC1	Mouse, monoclonal, IgG	1:1000	Millipore (OP80)
Cleaved caspase 3	Rabbit polyclonal, IgG	1:500	Cell Signaling (9661S)

### Fluorescence in situ hybridization (FISH)

FISH of murine *Epor* (RNAscope Probe-Mm-*Epor*) was performed in *Sox10-Cre*^*Tg/*+^*/EpoR*^*fx/fx*^ (cre+), and *Sox10-Cre*^+*/*+^*/EpoR*^*fx/fx*^ (cre-) animals at postnatal ages P7 and P11, using the RNAscope Multiplex Fluorescent Reagent Kit v2 from Advanced Cell Diagnostics and the Opal 520 fluorophore from Perkin Elmer as previously described in our protocol^28^. To quantify *Epors* on OLs, FISH was done followed by immunolabeling of Olig2 cells (see Table 2).

### Electron microscopy (EM)

Ultrathin tissue sections were prepared from P11 and P14 male mice for transmission electron microscopy (TEM) imaging, according to our previously described protocol^29^. Six animals per age and genotype were used for the analysis.

### Image acquisition for H&E, immunostaining, FISH, and EM analysis

Sections processed for H&E and MBP immunoperoxidase staining were analyzed with the MCID™ software, using calibrated images for normalization. Images were visualized and digitized using a precision illuminator (Northern Light Model B95, Imaging Research Inc., Brock University, St. Catharines, Canada) and Cool SNAP cf photo-camera (Photometrics, Tuscon, AZ, USA) with a Micro-Nikkor (55 mm + 12 mm) objective (Nikon Corp.).

All double and triple immunofluorescent and FISH stainings were imaged with a Zeiss LSM 700 or 900 confocal laser scanning microscope (Carl Zeiss AG, Oberkochen, Germany) with a 25X or a 40X oil immersion objective with a numerical aperture (NA) of 1.4, a scan zoom of 1X and an image size of 1024X1024 pixels. Immunofluorescence images from stratium and corpus callosum areas were taken as z-stacks (10–15 sections at 1 μm intervals).

FISH images from SVZ were taken as z-stacks (6 sections with 0.5 μm intervals). All images were recorded with sequential scanning to avoid overlapping of the emission spectra, and the confocal pinhole was set to 1 airy unit. Four different sections were taken per animal and condition. For statistical analysis, all sections were averaged per mouse.

The region of analysis for axonal myelination was the striatum. The areas of interest for OL differentiation were the subventricular zone of the third ventricle, the corpus callosum (CC), and the striatum. FISH samples were imaged in the subventricular zone and colocalized with Olig2 staining.

For EM, semithin sections of posterior white matter were observed with a bright field microscope. Then EM micrographs of the caudal white matter were taken with the 100 kV transmission electron microscope (TEM—Philips CM100 and Telos) connected to a digital CCD camera.

### Brain volume analysis

Data analysis was done using the MCID software (MCID Elite 6.0, InterFocus Imaging Ltd., Cambridge, UK). Four pups from different breeding pairs were analyzed per genotype and age in sagittal serial sections throughout one hemisphere. Serial sampling fraction (ssf) varies according to age, as specified in Table 1.

The total volumes (V_tot_) of the prosencephalon, dorsal pallium (cortex), middle pallium (hippocampus), and diencephalon (TH and HTH) were calculated from the areas delineated (Fig. 1D,^74^) in every section as follows:

$${V}_{tot}= ssf\times \sum_{i=1}^{n}{A}_{i}\times h$$

where: A_i_–A_n_; n = number of sections analyzed, and the section thickness (h)

### MBP optical density analysis

Using the MCID software (MCID Elite 6.0, InterFocus Imaging Ltd., Cambridge, UK), MBP immunoperoxidase staining intensity was assessed by densitometry analysis. Gray value calibration (Kodak step tablet no. 310ST607) was performed, and the intensity was measured in the different regions of interest: cortex, striatum, and CC. Each sample was corrected for background staining variations by subtracting the intensity value measured in an unmyelinated area.

### Immunofluorescence image analysis

The degree of axonal myelination was determined in double-immunofluorescence images for MBP and NF200 with the Software Imarisx64, 8.3.0, Bitplane AG, CH. (Center for Microscopy and Image Analysis, Zurich), by identifying the surfaces of NF200 and MBP through the “surface” function and setting the threshold and number of voxels to discriminate between signal and background noise. Subsequently, 3D figure reconstruction was done with the “wizard” function with surface and ratio automatically generated by the program. Once 3D reconstructions were done for both channels, the “surface co-localization” tool linked to Imaris Xtension was run, giving the surface-to-surface co-localization area covered by MBP over Nf200 normalized to the field of view.

For image analysis of OL proliferation, OL apoptosis, OL maturation, myelination (MBP), and *Sox10-*tdTom expression, maximum intensity projections from consecutive images of a z-stack were created using Fiji (ImageJ imaging software, National Institutes of Health, Bethesda, USA). Stack images from Olig2 and MBP single stains were converted to 8-bit greyscale before proceeding, and the threshold was manually subtracted to discriminate between signal and background noise. Then, total Olig2+ numbers per field of view were automatically counted with the Fiji Analyze Particles tool. Merged cells were cut by adding a 1-pixel thick line. Size particles were adjusted between 5-Infinity mm^2^ and circularity from 0.1 to 1.0. In the display, results were controlled so that each outline choice corresponded to one individual cell. MBP cells were quantified manually with the cell counter plugin. Double Ki67/Olig2 stained cells were identified with the Fiji colocalization threshold plugin. Before colocalization, color channels were made composite, and the background was subtracted manually for each channel. A new greyscale image, including the colocalized pixels above their respective thresholds, was created, and the colocalized cells were counted with the Fiji Analyze Particles tool as described for Olig2. Double NG2/Olig2 and CC1/Olig2 stains were quantified manually with the cell counter plugin. At least three serial sections of the SVZ and corpus callosum were quantified.

### FISH analysis

Analysis was done in z-stack images with maximum intensity projection using a custom-made cluster analysis macro in Fiji/ImageJ (NIH) software. Processing of *Epor* particles (green channel) was separately analyzed, with background subtraction using rolling ball radius, Gaussian blurring, and thresholding for selecting regions of interest of high staining intensity, as well as shape (0.5–1 circularity) and size (0.1–1 μm diameter) restrictions. The same parameters were used in all images per genotype and age. Four animals and three images per animal were used for the analysis across brain areas. Colocalization of *Epors* with Olig2+ stained cells was identified with Fiji. Once *Epor* particles (green channel) were processed as previously described, a new merged image with both channels was made. Olig2+ cells (magenta channel) were identified based on the nucleus centroid and an area of a nucleus-cytoplasm ratio of 0.6 was selected for quantification of the *Epor* particles (Supplementary Fig. 3). *Epor* dots were made binary and counted with the Analyze Particles tool with shape (0.5–1 circularity) and size (0.1–1 μm diameter) restrictions.

### Electron microscopy (EM) analysis

Axon diameter and myelin sheet thickness were determined in images from electron microscopy captured with 17,500× magnification. Axon diameter was the average of the largest and smallest diameter, and myelin sheet thickness was determined by the number of turns around the axon and the g-ratio (g) = di/do, where: di = axon diameter without myelin; do = total axon diameter.

### Western Blot fluorescent analysis

Brain tissue samples were collected from *Sox10-Cre*^*Tg/*+^*/EpoR*^*fx/fx*^ (cre+) and *Sox10-Cre*^+*/*+^*/EpoR*^*fx/fx*^ (cre-) mice at P4, and P11. Animals were deeply anesthetized with an intraperitoneal injection of sodium pentobarbital (50 mg/kg; Nembutal, Kantonsapotheke Zürich) followed by decapitation and dissection of brain tissue on ice. Tissues containing the midbrain, striatum, and SVZ were dissected and placed on ice. Samples from each animal were independently processed (n = 4 animals/group). Total tissue was homogenized by passing it through a 24 G needle several times in ice-cold 300 µl RIPA buffer (50 mM Tris/HCl pH 8.0, 150 mM NaCl, 1% NP-40, 0.5% Na deoxycholate, 1 mM EDTA, 0.1% SDS) with Protease Inhibitor Cocktail Set III, EDTA-Free diluted 1:100 (Merck Millipore, #539134), 1 mM Sodium orthovanadate, and 20 mM sodium fluoride as tyrosine and serine/threonine phosphatases inhibitors. Samples were kept on ice for 30 min and whole cell lysates were collected after centrifugation at 13,000 rpm for 20 min at 4 °C. Protein concentrations were determined using a Pierce BCA assay (Thermo Scientific, #23228, #23224). Stock samples were prepared in a 5 × Laemmli buffer (4% SDS, 20% glycerol, 10% mercaptoethanol, 0.004% bromophenol blue, and 0.125 M Tris HCl pH 6.8) by boiling at 70 °C for 5 min. Protein samples (30 µg) were run in 10% SDS-PAGE gels for 120 min at 25 mA. Samples were then transferred for 1 h 40 min at 120 V to a 0.45 µm nitrocellulose blotting membrane (GE Healthcare, #10600002) using a TE42 Transfer tank unit with the cooling chamber (Hoefer™, USA). Membranes were washed 2 × for 10 min with 0.05% TBST followed by blocking in 5% bovine serum albumin (BSA) in 0.05% TBST for 1 h at room temperature. Membranes were incubated at 4 °C overnight with the following primary antibodies diluted 1:1000 in 5% BSA in 0.05% TBST solution: mouse anti p44/42 MAPK (Erk1/2) (Cell Signalling, #9107); rabbit anti-Phospho-p44/42 MAPK (p-Erk1/2) (Cell Signaling, #9101). Membranes were washed and incubated in secondary antibody solutions conjugated to fluorescent molecules for two-color detection of Erk1/2 and phospho Erk1/2 proteins (anti-rabbit IRDye 680 LT red and anti-mouse IRDye 800CW green, respectively), diluted 1:8000 in 5% BSA in 0.05% TBST blocking solution for 1 h at room temperature. Protein loading was controlled with a 1:1000 anti-vinculin antibody (Abcam, #130007). The PageRuler™ prestained protein ladder 10 to 170 kDa (26616, Thermo Scientific) was used for protein sizing.

Detection of fluorescent bands was completed with the Li-Cor Odyssey Platform (LI-COR Biosciences). Proteins were analyzed densitometrically with ImageJ (NIH) software. Band intensities from AKT and Erk1/2 were corrected with band values determined on vinculin.

### RNA preparation and quantitative real-time PCR (RT-qPCR)

Tissue containing the striatum and the subventricular zone was collected from our two transgenic mice lines and controls at P4 and P11: N = 8 male animals per age in Tg21 and N = 5 per age in *Sox10*-cre;*EpoR*^*fx/fx*^ mice line. Total RNA was extracted using the ReliaPrep RNA Tissue Miniprep System (Promega, #Z6110) according to the manual. DNase I digestion was used to eliminate DNA contamination. RNA purity and quantity were determined spectrophotometrically (Nanodrop 2000, ThermoScientific). First-strand cDNA was obtained by reverse transcription using the RevertAid First Strand cDNA Synthesis Kit (ThermoFisher Scientific, #K1622). Samples (10 ng/µl cDNA) were analyzed by SYBR Green (ThermoFisher Scientific, #A25741) semi-quantitative real-time PCR (qRT-PCR) (7500 Fast Real-Time PCR System, ThermoFisher Scientific). Primers for mRNA expression analyses were designed with Primer 3.0. Software to amplify human or murine genes without cross-specificity (see Table 3). Oligo properties were calculated using Oligo Analyzer 3.1. mRNA expression levels were calculated using the ∆∆Ct method^75^ and normalized to beta-actin (*βACT)*. Each group of samples was normalized to WT P4.

**Table 3:** Primers used for RT-qPCR.

*Nfatc1*	*F: 5′-GGAGCGGAGAAACTTTGCG-3′* *R: 5′-GTGACACTAGGGGACACATAACT-3′*
*Nfatc2*	*F: 5′-CCACCACGAGCTATGAGAAGA-3′* *R: 5′-GTCAGCGTTTCGGAGCTTCA-3′*
*Nfatc3*	*F: 5′-GCTCGACTTCAAACTCGTCTT-3′* *R: 5′-GATGTGGTAAGCCAAGGGATG-3′*
*Nfatc4*	*F: 5′-GAGCTGGAATTTAAGCTGGTGT-3′* *R: 5′-GGAGGGGTATCCTCTGAGTCC-3′*
*Ppp3ca*	*F: 5′-GTGAAAGCCGTTCCATTTCCA-3′* *R: 5′-GAATCGAAGCACCCTCTGTTATT-3′*
*Ppp3cb*	*F: 5′-CGCGTCGTCAAAGCTGTTC-3′* *R: 5′-CCTGGGTATCCCATCCATATCAA-3′*
*Ppp3cc*	*F: 5′-ATGCCACCCCGAAAAGAGG-3′* *R: 5′-CATGGTCGGTCCTTCTTGACG-3′*
*Ppp3r1*	*F: 5′-ATGGGAAATGAGGCGAGTTACC-3′* *R: 5′-TCCACGCTCAAAGAACCAGAA-3′*
*Ppp3r2*	*F: 5′-ATCGGGCTCCCTGAGCATA-3′* *R: 5′-TCGAAGATGTCGATCACTCGG-3′*
*Nkx2.2*	*F: 5′-ATGTCGCTGACCAACACAAAG-3′* *R: 5′-GCTGTCGTAGAAAGGGCTCTT-3′*
*Nkx6.2*	*F: 5′-GCATGACCGAGAGCCAAGT-3′* *R: 5′-GCATCCGAGTCTTGCTTCTTTTT-3′*
*Sox10*	*F: 5′-CGGACGATGACAAGTTCCCC-3′* *R: 5′-GTGAGGGTACTGGTCGGCT-3′*
*Myrf*	*F: 5′-TCTGGGCCTCCCATCAAAG-3′* *R: 5′-CGGGGTTATGGTGCGTAGAAG-3′*
*βACT*	*F: 5'-TTT CCA GCC TTC CTT CTT GGG-3'* *R: 5'-GAGGTCTTTACGGATGTCAACG-3'*

### Developmental milestone tests

We evaluated the postnatal development of mice following a battery of tests examining weight gain, physical development, the appearance of reflexes, and motoric development throughout the first 21 days of life^76,77^.

*Physical Appearance* includes skin pigmentation, fur appearance, incisor eruption eye-opening. *Reflex Appearance* consists of the righting reflex and cliff avoidance in which the vestibular and locomotor systems and the body strength and coordination of the animals are evaluated; negative geotaxis, which evaluates the vestibular/labyrinthine system of the animals; and vestibular drop, in which the vestibular system and body strength are tested when the animal is suspended by its tail and raises its head to the level of the hind limbs by arching its body sideways. All these reflexes were tested for 30 s in N = 10 mice per genotype. *Motoric development* was evaluated by monitoring the age at which the mice started pivoting, crawling, and walking. The open field test evaluates the speed of quadrupedal locomotion in a circle with a diameter of 13 cm, and the time needed to leave the circle was registered. The maximum given time for the test was 30 s. Tests were done on 6 to 15 animals per genotype.

### Motor coordination and motor learning

Using the rotarod performance test, based on a rotating rod suspended above a cage floor, high enough to induce avoidance to fall but not injury, we tested motoric coordination at P14, P21, and P60, and motoric learning at P21. Motoric coordination was tested in P14 mice four times for 4 min with 5 min recovery intervals. The initial rotation speed was set to 1 revolution per min (rpm), with an acceleration of 10 rpm within 4 min. Motoric coordination in P21 and P60 mice was tested four times for 5 min with 5 min recovery intervals. The initial rotation speed was 4 rpm and acceleration to 45 rpm within 5 min. An activity control test was performed four times with a constant speed of 10 rpm for 5 min. The time to fall and speed were recorded in each test. Tests were done on 8 to 20 animals per genotype and age. Tests were done on 10 to 20 animals per genotype and age.

### Experimental design and statistical analyses

Data collection was conducted by laboratory members who were blinded to the genotypes. Tissues from mice were collected at postnatal ages (P): 3, 7, 11, 14, 21, and 25 for morphological evaluation of brain growth, myelination, and OL precursor cell (OPCs) differentiation and maturation. In addition, tissue was collected at P9, P11, and P14 for ultrastructural evaluation of myelin ensheathment with electron microscopy. Fresh tissue was also collected at P7 for *Epors* expression analysis, at P4 and P11 for pro-differentiation and pro-myelination transcript analysis, and WB analysis in Sox-cre mice. No more than one male pup per age was taken from each breeding group. Six different breeding groups were used per genotype, and their controls for behavioral analysis, and tissue was collected afterward.

Statistical analyses and graphs were performed using GraphPad Prism 9.0.0 (GraphPad Software, San Diego, CA, USA). We used scatterplots with bars to present single data points and the mean ± standard deviation (SD). Parametric data with one condition (genotype) were analyzed using an unpaired, two-tailed Student’s t-test. A two-way ANOVA with multiple comparisons post hoc test was used to compare data influenced by two factors (e.g., age and genotype or brain areas and genotype). Normality was tested with Kolmogorov–Smirnov (KS) test. The difference in relative frequency distributions between WT and Tg21 was tested with a 2-sample Kolmogorov–Smirnov (2 s-KS) test using SPSS 28.0.1 (IBM). A P value < 0.05 was considered statistically significant for all statistical tests. To confirm appropriate sample sizes for experiments, a power analysis was conducted using G*Power software (Heinrich Heine Universität, Düsseldorf, Germany)^78^. The F ratio and the exact *P* value for multiple comparisons are described in the figure legends.

### Supplementary Information

Supplementary Figures.

## Data Availability

The data that support the findings of this study are available from the corresponding author upon reasonable request.
