# Supplementary material for: Erythropoietin regulates developmental myelination in the brain stimulating postnatal oligodendrocyte maturation
Source: Sci Rep. 2023 Nov 9;13:19522. doi: 10.1038/s41598-023-46783-9 (PMC10636124; doi:10.1038/s41598-023-46783-9)
Supplement: Supplementary file 1 — Supplementary Figures. [file 41598_2023_46783_MOESM1_ESM.pdf]

Erythropoietin regulates developmental myelination in the brain stimulating postnatal oligodendrocyte maturation

Paola Muttathukunnel[1,2,*], Michael Wälti[1,*], Mostafa A. Aboouf[3,4,5], Christina Köster-Hegmann[1,3], Tatjana Haenggi[1], Max Gassmann[3,4], Patrizia Pannzanelli[6], Jean-Marc Fritschy[1,2], and Edith M. Schneider Gasser[1,2,3,+]

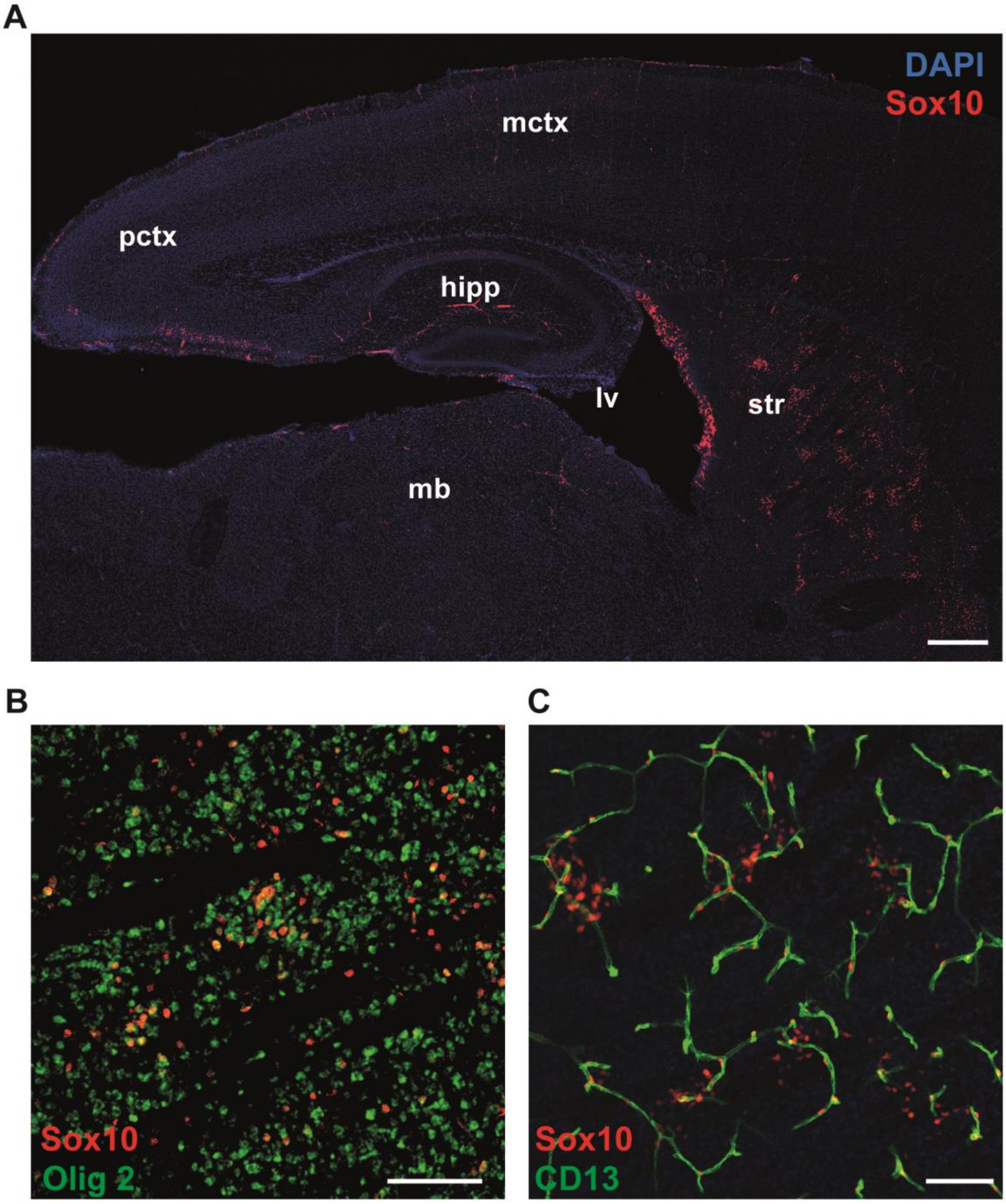

Supplementary Figure S1. Sox10-tdTom cell expression in the brain at postnatal day 7. **(A)** Confocal tiled images (10X) of a sagittal brain cut showing the posterior cortex (pctx), hippocampus (hipp), medial cortex (mctx), striatum (str), lateral ventricle (lv), and midbrain. Sox10-tdTom cells are highly expressed in the lv and str. **(B)** Confocal tiled images (10X) of the striatum showing a high proportion of Sox10-td-Tom cells colocalized with Olig2 immunostained cells. **(C)** Confocal tiled images (10X) of the striatum with a small portion of Sox10-tdTom cells colocalized with CD13, a pericyte marker. Scale bars: (A) 400 μm, (B) 100 μm, (C) 100 μm.

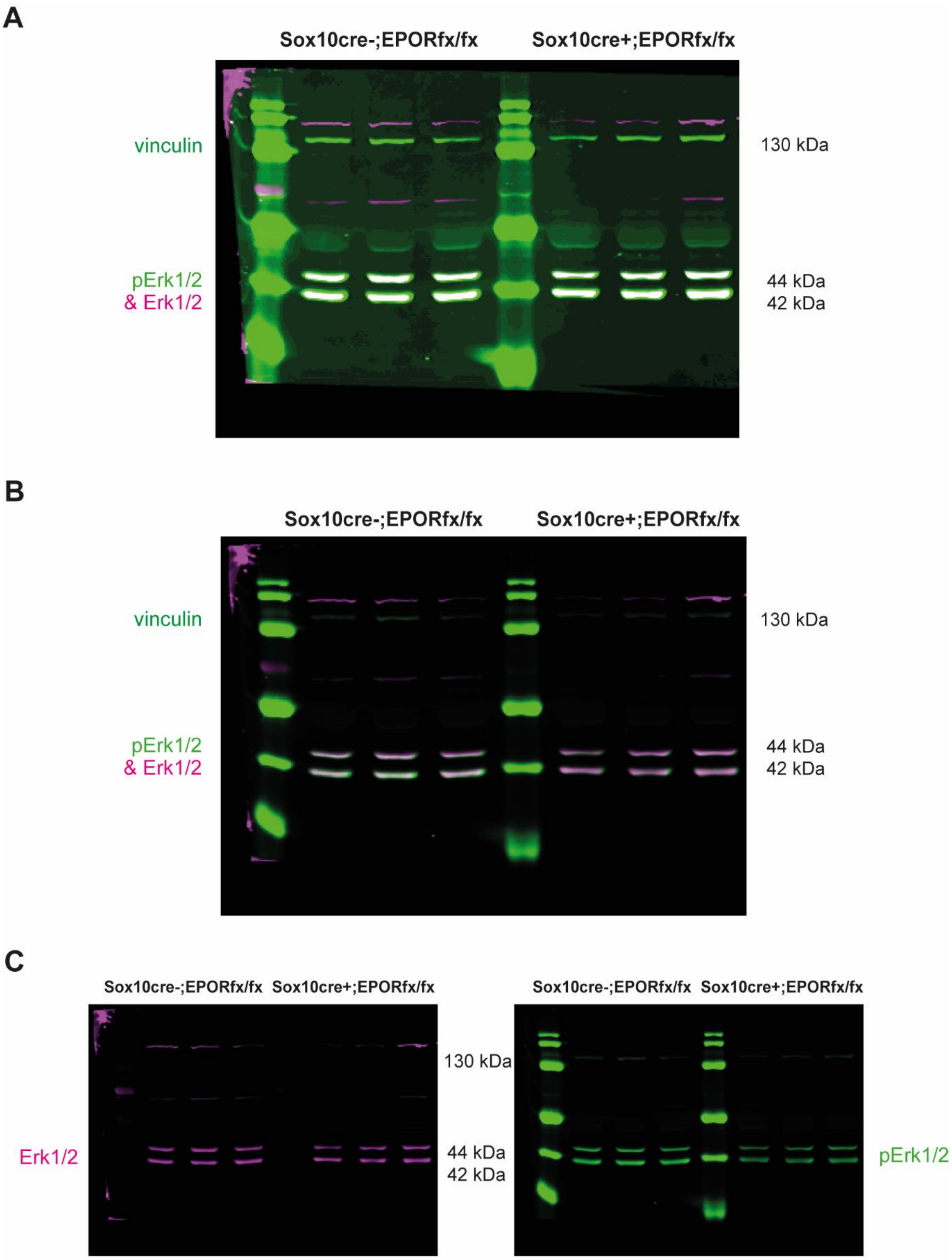

Supplementary Figure S2. Fluorescent western blot gel for Erk1/2 (magenta) and phosphorylated Erk1/2 (green) and vinculin (green). **(A)** Overexposed blot to see the gel edges and vinculin. **(B)** Original autoexposure gel with both channels. **(C)** Original autoexposure gel separate channels.

A

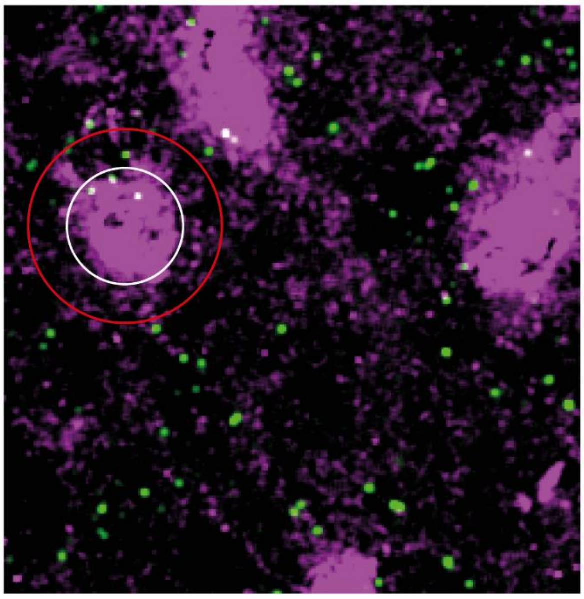

B

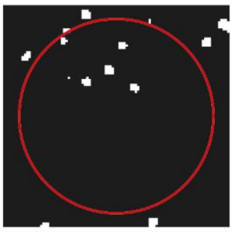

Supplementary Figure S3. Example of quantification of *Epors* on Olig2+ cells. **(A)** Merged images with Olig2+ cells (magenta channel) and *Epor* (green channel). Olig2+ cells are identified based on the nucleus centroid. The nucleus area (white) is measured, and a nucleus to the cytoplasm ratio of 0.6 (red) is selected for quantification (area=nucleus/0.6). **(B)** *Epor* dots are made binary and counted with the Analyze Particles tool with shape (0.5-1 circularity) and size (0.1–1 um diameter) restrictions.
